# Extracellular signal-regulated kinase 5 increases radioresistance of lung cancer cells by enhancing the DNA damage response

**DOI:** 10.1038/s12276-019-0209-3

**Published:** 2019-02-21

**Authors:** Weiwei Jiang, Guanghui Jin, Fangfang Cai, Xiao Chen, Nini Cao, Xiangyu Zhang, Jia Liu, Fei Chen, Feng Wang, Wei Dong, Hongqin Zhuang, Zi-Chun Hua

**Affiliations:** 1 The State Key Laboratory of Pharmaceutical Biotechnology, College of Life Sciences, Nanjing University, Nanjing, PR China; 2 Department of Basic Medical Sciences, Medical College, Xiamen University, Xiamen, PR China; 3 Department of Nuclear Medicine, The Affiliated Nanjing First Hospital, Nanjing Medical University, Nanjing, PR China; 4 Changzhou High-Tech Research Institute of Nanjing University and Jiangsu Target Pharma Laboratories Inc., Changzhou, 213164 PR China

**Keywords:** Lung cancer, Radiotherapy

## Abstract

Radiotherapy is a frequent mode of cancer treatment, although the development of radioresistance limits its effectiveness. Extensive investigations indicate the diversity of the mechanisms underlying radioresistance. Here, we aimed to explore the effects of extracellular signal-regulated kinase 5 (ERK5) on lung cancer radioresistance and the associated mechanisms. Our data showed that ERK5 is activated during solid lung cancer development, and ectopic expression of ERK5 promoted cell proliferation and G2/M cell cycle transition. In addition, we found that ERK5 is a potential regulator of radiosensitivity in lung cancer cells. Mechanistic investigations revealed that ERK5 could trigger IR-induced activation of Chk1, which has been implicated in DNA repair and cell cycle arrest in response to DNA double-strand breaks (DSBs). Subsequently, ERK5 knockdown or pharmacological inhibition selectively inhibited colony formation of lung cancer cells and enhanced IR-induced G2/M arrest and apoptosis. In vivo, ERK5 knockdown strongly radiosensitized A549 and LLC tumor xenografts to inhibition, with a higher apoptotic response and reduced tumor neovascularization. Taken together, our data indicate that ERK5 is a novel potential target for the treatment of lung cancer, and its expression might be used as a biomarker to predict radiosensitivity in NSCLC patients.

## Lung cancer: Understanding radiotherapy resistance

Resistance to radiotherapy in patients with lung cancer may be countered by targeting a protein involved in promoting DNA repair. Radiotherapy causes DNA double-stranded breaks in lung cancer cells in order to kill them. However, cancer cells can show improved DNA repair and responses to damage, resulting in resistance to treatment. Zi-Chun Hua, Hongqin Zhuang at Nanjing University in China and co-workers examined the activity of the extracellular signal-related kinase 5 (ERK5) protein in response to the stress of ionizing radiation. They found that after radiation exposure ERK5 increased expression of another protein involved in DNA repair, facilitating cancer cell recovery. Knocking out ERK5 suppressed this resistance to radiotherapy. ERK5 could be a valuable target for treating lung cancer, and ERK5 expression level could be used as a biomarker for patient sensitivity to radiotherapy.

## Introduction

Lung cancer is currently the leading cause of cancer-related death in both developing and developed countries. Non-small cell lung cancer (NSCLC) is responsible for >80% of all lung cancer cases^1^. Radiotherapy plays an important role in curative treatment of patients with advanced and inoperable NSCLC^2^. Nevertheless, radioresistance has become a serious obstacle limiting the clinical benefits of radiotherapy. Many mechanisms potentially responsible for radiotherapeutic resistance have been extensively studied. However, the exact mechanisms are still unclear, likely due to tumor heterogeneity and the various factors involved.

In general, DNA double-stranded breaks (DSBs) are the major cytotoxic lesion resulting from ionizing radiation (IR) and can lead to genome instability and cell death. Nevertheless, upon IR, cancer cells exhibit enhanced DNA damage response and DNA repair capacity, which reduces the extent of radiation-induced damage and resultant death. The DNA damage signaling response is regulated by ataxia telangiectasia-mutated (ATM) and ATM- and Rad3-related (ATR) kinases, which belong to the phosphoinositol 3-kinase-like kinase (PIKK) family^3–5^. Once activated, the histone variant H2AX and a subset of downstream effectors, such as the cell cycle checkpoint kinases (Chk1 and Chk2), are phosphorylated by ATM and ATR, leading to the activation of cell cycle checkpoints and induction of cell cycle arrest. Therefore, suppressing the DNA damage response and decreasing the DNA repair capacity in tumor cells might potentially overcome resistance to radiation.

Mitogen-activated protein kinases (MAPKs) are a group of conserved protein Ser/Thr kinases that play important roles in intracellular signal transduction, such as gene expression, cell proliferation, cell motility, cell survival, and death^6,7^. Three well-characterized MAPK subfamilies have been found, namely, p38, the Jun N-terminal kinases (JNKs)/stress-activated protein kinases (SAPKs), and the extracellular signal-regulated kinases (ERK1/2)^8,9^. Extracellular signal-regulated kinase 5 (ERK5), also known as big MAP kinase 1 (BMK1), is the most recently identified and least studied mammalian MAP kinase cascade. It is activated by growth factors, hyperosmotic shock, oxidative stress, laminar flow shear stress, and UV irradiation^10–13^. Recently, major progress has been made in understanding the regulation of ERK5 and its functions. For example, inactivation of ERK5 resulted in angiogenic failure and cardiovascular defects leading to embryonic lethality around embryonic days 9.5–10.5^14,15^, indicating that ERK5 has certain biological functions in angiogenesis and cardiac development. Furthermore, ERK5 plays essential roles in the maintenance of vascular integrity and tumor-related angiogenesis, likely through its capacity to phosphorylate rpS6 in endothelial cells^16^. ERK5 has been found to be associated with cancer due to its abnormal expression in human tumors^17^. Similar to the other MAPK families, ERK5 signaling is activated by many oncogenes^17^, e.g., by the oncogene Ras in certain cell types, including PC12, C2C12, and COS7 cells^18–20^. Constitutively activated ERK5 was also reported to be associated with activated forms of ErbB2, ErbB3, and ErbB4 in human breast cancer cells and are related to shorter disease-free intervals, poor prognosis, resistance to chemotherapy, and increased risk of metastasis^21–23^. These findings indicate that ERK5 signaling activation could be involved in the carcinogenesis process and that ERK5 might be a potential molecular target for several tumor therapies.

In this study, we investigated the role of ERK5 in NSCLC radioresistance and reveal that knockdown of ERK5 expression suppressed while increased ERK5 expression promoted radioresistance in lung cancer cells. Moreover, we demonstrate that ERK5 facilitates Chk1 phosphorylation induced by IR. Thus, we speculated that ERK5 downregulation can decrease radioresistance and might have high translational potential. As expected, our data suggested that ERK5 siRNA or the ERK5 inhibitor XMD8-92 preferentially increased NSCLC cell sensitivity to IR treatment and enhanced the radiotherapeutic index of lung cancer.

## Materials and methods

### Materials

An Annexin V-FITC kit was obtained from BD Biosciences. Antibodies against ERK5, phospho-ERK5, ERK1/2, phospho-ERK1/2, phospho-checkpoint kinase 1 (Chk1), phospho-checkpoint kinase 2 (Chk2), phosphor-ATM kinase, phosphor-ATR protein, cleaved caspase-9, cleaved caspase-8, cleaved caspase-3, Cyclin B1, Cdc-2, and Cdc25C were purchased from Cell Signaling Technologies. Antibodies against p53, α-tubulin, β-actin, and cleaved poly (ADP-ribose) polymerase (PARP), and the ERK5 inhibitor XMD8-92 were purchased from Santa Cruz Biotechnology. Horseradish peroxidase (HRP)-conjugated goat anti-rabbit and anti-mouse IgG antibodies were also obtained from Santa Cruz Biotechnology. Anti-H2A histone family, member X (H2AX, Ser139) was provided by Millipore (Billerica, MA, USA). Alexa Fluor 488 goat anti-mouse IgG (H+L) secondary antibody was purchased from Invitrogen (Carlsbad, CA, USA). Anti-CD31 antibody was obtained from Abcam (Cambridge, MA, USA). Sources of other materials are noted accordingly in the study.

### Cell culture and transfection

The NSCLC cell lines A549 and H1299, mouse Lewis lung cancer cell line LLC, mouse embryonic fibroblast cell line NIH 3T3, mouse melanoma cell line B16, mouse leukemic monocyte macrophage cell line RAW 264.7, and human bronchial epithelial cell line BEAS-2B were purchased from the ATCC (Philadelphia, PA, USA). The cells were cultured in Dulbecco’s modified Eagle’s medium (DMEM) or RPMI 1640 (Invitrogen, Carlsbad, CA, USA), supplemented with 10% (v/v) fetal bovine serum (FBS; Invitrogen, Carlsbad, CA, USA) and 1% penicillin–streptomycin (Invitrogen, Carlsbad, CA, USA). All cells were cultured in a humidified CO_2_ incubator at 37 °C. Plasmids were introduced into cells via polyethylenimine (PEI)-mediated transfection as described previously^24^. Briefly, cells were seeded at a density of 2 × 10^6^ cells/10 cm dish (Corning, Lowell, MA, USA) and were transfected with PEI-complexed plasmids (at a ratio of 2:1, w/w) in serum-free DMEM. Two hours after addition of the DNA, the medium was replaced with fresh complete medium, and transfected cells were continuously cultured until harvested for analysis.

### Plasmids, shRNA, and establishment of stably transfected cell lines

ERK5 cDNA was amplified using cDNA prepared from A549 cell-extracted mRNA as a template and subcloned into a pcDNA3.1-V5 vector (Invitrogen, Carlsbad, CA, USA). Cell transfections were conducted with M-PEI reagent as described previously^24^. Short hairpin RNAs (shRNA) against mouse ERK5 were designed by using a web-based shRNA design program (http://www.genscript.com), and double-stranded oligonucleotides containing the specific sequences for open reading frames of the genes were cloned into a pRNAT-U6.1/Neo vector (GenScript, Piscataway, NJ, USA). We designed three different shRNA constructs to target ERK5. The target sequences are listed in Table S1. shRNA against the luciferase vector pRNAT-U6.1/Hygro/siFLuc (GenScript) was used as the control. For the establishment of a stably transfected cell line, A549 cells were transfected with the pcDNA3.1-V5-ERK5 construct. The stably expressing transfected cell lines were obtained by selecting the transfectants using G418 (500 µg/mL). Immunoblotting analysis was used to further confirm cognate protein expression in all the transfectants.

### Soft agar assay

For this assay, a base layer of 10% FBS/DMEM containing 0.7% agarose was spread in a 60-mm plate; 2 × 10^4^ cells were resuspended in 3 mL of 10% FBS/DMEM with 0.4% agarose as the upper layer. The plates were incubated at 37 °C with 5% CO_2_. After 3 weeks of growth, cell colonies were stained with crystal violet and counted. Each experiment was performed in triplicate, as previously indicated^25^.

### Colony formation assay

Cells were seeded in 60-mm plates (approximately 500 cells/plate). After 24 h, the cells were treated with different doses of radiation and/or ERK5 siRNA or the ERK5 inhibitor XMD8-92 (5 µM) and then cultured for 12 days. The formed colonies were fixed with methanol for 15 min and stained with a 1:10 dilution of Giemsa reagent (Merck, Germany) for 10 min. Survival fractions were calculated by normalization to the plating efficiency, which was obtained by dividing the average number of colonies by the number of cells plated, in the appropriate control groups. The dose–enhancement ratio (DER) was calculated by dividing the radiation dose without ERK5 siRNA by the radiation dose with ERK5 siRNA required to achieve the same degree of cell death. If the DER was <1, the agent was considered radioprotective, and if the DER was >1, the agent was considered radiosensitizing. The data were fitted to the linear quadratic model using GraphPad Prism-5 software (GraphPad Prism Software Inc., La Jolla, CA, USA), as previously described^26^.

### Immunofluorescence assay (IFA)

The immunofluorescence assay was performed as previously indicated^27^. A549 cells were irradiated with 5 Gy of X-rays and incubated for 8 h after IR treatment. The cells were harvested and immunostained with anti-histone H2AX phosphorylation (γH2AX) antibody. Then, the cells were incubated with Alexa Fluor 488-labeled (Thermo Fisher Scientific Inc.) secondary antibody for 1 h at room temperature. Images were acquired using an Olympus laser scanning confocal microscope (Olympus Optical Co., Tokyo, Honshu, Japan). For each treatment condition, the number of fluorescently labeled γH2AX foci was assessed via fluorescence microscopy in at least 50 cells.

### Cell proliferation and apoptosis assay

Cells transfected with both empty and ERK5 expression vectors were seeded in 96-well culture plates at a density of 2000 cells per well. The cells were incubated for 2, 3, or 4 days, and cell viability was determined with an MTT assay at 570 nm using a microplate reader (Bio-Tek Instruments, Winooski, VT, USA). For apoptosis assays, cells were seeded in 6-well culture plates at a density of 5 × 10^5^ cells per well and treated with 5.0 Gy of IR and/or ERK5 siRNA or the ERK5 inhibitor XMD8-92 (5 µM). After 24 h, the cells were trypsinized, washed twice with cold PBS, and resuspended in 100 µL binding buffer. Annexin V-FITC (0.1 µg) and propidium iodide (PI, 10 µL, 50 µg/mL) were added according to the manufacturer’s instructions (BD Biosciences, CA, USA), and the stained cells were analyzed with a FACSCalibur flow cytometer (BD, Denderstraat, Belgium) and CellQuest software within 1 h, as previously described^28^.

### Cell cycle analysis using flow cytometry

Cell cycle distribution was analyzed via flow cytometry using a BD FACSCalibur flow cytometer (BD Biosciences), and the data were analyzed with CellQuest software (BD Biosciences, CA, USA), as described in earlier studies^29^.

### Western blotting and enzyme-linked immunosorbent assay (ELISA)

Whole-cell lysates of A549 and H1299 cells (approximately 5 × 10^6^ cells) were prepared with RIPA buffer (Santa Cruz Biotechnology)^30^ containing PMSF, orthovanadate, and protease inhibitors. Protein concentration was determined with a Bradford assay (Bio-Rad Laboratories). Equal amounts of protein (50 µg) were separated in 10% SDS-PAGE gels. After protein transfer to a nitrocellulose membrane and blocking with 5% milk in TBS buffer, the protein of interest was immunoplexed with the indicated primary antibody and corresponding secondary antibody. Bound antibodies were then visualized with ECL plus western blotting detection reagents (GE Healthcare). Signal intensity was quantified by densitometry using ImageJ software (NIH, Bethesda, MD, USA). The ELISA for VEGF was performed by using a VEGF ELISA kit (Boster, Wuhan, China) according to the manufacturer’s instructions. All experiments were prepared in triplicate and performed at least three times independently.

### RNA extraction and quantitative real-time PCR

Total RNA was extracted from cells using a TRIzol Kit (Invitrogen, Carlsbad, CA, USA), and 1 μg was used for cDNA synthesis primed with Oligo(dT)18 primers (Takara, Dalian, China). Then, qPCRs were carried out according to the manufacturer’s instructions using SsoFast EvaGreen Supermix on a CFX96 Real-Time System (Bio-Rad Laboratories, Hercules, CA, USA); the oligonucleotide primers are shown in Table S2. Each qPCR reaction was repeated at least 3 times, and β-actin was used as the internal control, as previously described^28^.

### Luciferase assay

A549 cells were cotransfected with p53-Luc plasmid (Agilent Technologies, Santa Clara, CA, USA) and pRL-TK Renilla vector (Promega, Madison, WI, USA) using Lipofectamine 2000 reagent (Invitrogen, Carlsbad, CA, USA). After 24 h, the transfected cells were treated with passive lysis buffer at room temperature for 10 min. Cell lysates were then transferred to a plate, and luciferase assay reagent and stop reagent were added in sequence. Next, luciferase activity was measured using a Luciferase Assay System (Promega, Madison, WI, USA) according to the manufacturer’s instructions, as previously described^31^.

### In vivo tumor xenograft study

Athymic nude mice (6–8 weeks of age) were obtained from Shanghai Laboratory Animal Center (Shanghai, China) and housed under germ-free conditions. Female C57BL/6J mice (Vital River, Beijing, China) were housed in a temperature-controlled sterile room where humidity and light were carefully monitored. Animal welfare and experimental procedures were in strict accordance with high standard animal welfare and other related ethical regulations approved by Nanjing University Animal Care and Use Committee. Animal tumor experiments and PEI-mediated gene transfection were performed as described with certain modifications^24^. Briefly, tumors were generated in nude mice and C57BL/6J mice via subcutaneous (s.c.) injection of A549 (2 × 10^6^ cells in 100 µL PBS) or LLC cells (1 × 10^6^ cells in 100 µL PBS) into the right hind leg of each mouse. Tumor measurement was converted to tumor volume (*V*) using the formula *L* × *W*^2^ × 0.52, where *L* and *W* are the length and width, respectively. When tumors reached a size of 50–100 mm^3^, the mice were arbitrarily assigned to different groups to receive intratumoral injections of 40 µL M-PEI-complexed siERK5 or siCtrl as a control. Plasmids (8 µg per injection) were injected into each animal. The delivery efficiency of plasmids to tumor tissues has been described in a previous report^24,32^. Local radiotherapy (RT) was carried out with a deep X-ray machine (Model X.S.S.205 FZ, China) with 200 kV/10 mA using filters of 0.5 mm Cu/0.5 mm Al at a dose rate of 0.287 Gy/min, and control mice were sham-exposed^30^. In the other set of experiments, A549 cells were subcutaneously injected as above. When tumors reached a size of approximately 50 mm^3^, the mice were treated intraperitoneally twice a day for 24 days with XMD8-92 (25 mg/kg), local irradiation (6 Gy, fractionally administered on days 0, 2, and 4) or both. The antitumor activity of treatments was evaluated by assessing tumor growth inhibition. The tumors were collected and weighed at the end of the study.

In a parallel animal assay (a total of 4 groups, with 3 mice per group), the tumor establishment and treatment protocols were the same as described above. The mice were euthanized on the 25th day. Tumors were collected, fixed with 4% formaldehyde, embedded in paraffin, and sectioned for hematoxylin and eosin (H&E) staining according to standard histological procedures. The TUNEL technique was used to visualize apoptotic cells in tumor sections according to the manufacturer’s instructions (Vazyme, Nanjing, China).

### Immunohistochemistry (IHC)

The methods used for IHC have been described previously^30^. Briefly, paraffin-embedded tumor tissues were collected from LLC lung cancer-bearing mice and processed for sectioning (4 μm thick). Sections were incubated with an affinity-purified anti-VEGFR2 antibody (Cell Signaling) for 2 h. Bound antibody was detected with polymerized HRP anti-rabbit IgG (Maixin, Fuzhou, China) using diaminobenzidine tetrahydrochloride (DAB) as the substrate.

### Statistical analysis

Statistical analysis was carried out using SPSS software (version 11.0; SPSS, Chicago, IL, USA). The data are expressed as the mean ± standard deviation (SD). For multiple comparisons, statistical analyses were performed using one-way analysis of variance (ANOVA) with a Tukey post-test. For paired data, statistical analyses were performed using two-tailed Student’s *t*-tests. For all analyses, *p* < 0.05 was considered statistically significant.

## Results

### ERK5 expression is upregulated in solid tumor development

ERK5 is regulated by a wide range of mitogens and cellular stresses and regulates numerous physiological processes during development and pathogenesis^11–13,33^. For example, ERK5 overexpression in PC12 cell is correlated with the survival response to oxidative stress^33^. However, it is unclear whether ERK5 is expressed in various tumor cells and whether its expression changes during solid tumor development. Therefore, we chose NIH 3T3 cells and several cancer cell lines, namely, LLC, RAW, B16F1, and B16F10 cell lines, to observe ERK5 mRNA expression via qPCR analysis. Simultaneously, we assessed the levels of other certain molecular markers, such as HIF-1α and GRP-78, which are correlated with tumor progression in several types of cancer. As shown in Figure S1a, the ERK5 and GRP-78 mRNA levels were easily detected by qPCR in 3T3 cells and other tumor cell lines under normal culture conditions. The amount of ERK5 mRNA expression was variable, with the highest expression in LLC cells. However, HIF-1α mRNA expression was only detected in the above tumor cell lines, not in 3T3 fibroblast cells, at the determined PCR cycles (28 cycles). We next chose LLC cells to detect the expression of stress-related genes, including ERK5, in solid lung cancer growth progression. First, we generated LLC tumor-bearing mice. When tumor sizes reached approximately 400 mm^3^, mRNA was extracted from the LLC tumor cells as described in Materials and methods, and as a control, mRNA was also extracted from LLC cell lines cultured under normal conditions. As shown in Figure S1b, similar to the expression characteristics of stress-related genes (HIF-1α, HSP70, and GRP-78) and angiogenetic stimulators (VEGF and VEGF-C), the ERK5 mRNA level was obviously upregulated under solid lung cancer conditions. Additionally, we generated A549 (human lung adenocarcinoma epithelial cell line) tumor-bearing mice, and similar results were observed, as shown in Figure S1b. Next, the protein level of ERK5 was detected by western blotting. Consistent with the qPCR results, both the total protein level and the phosphorylation status of ERK5 were considerably elevated in LLC and A549 solid tumors (Figure S1c). We further validated the increased expression of ERK5 in human lung adenocarcinoma tissues compared with normal lung tissues by using The Cancer Genome Atlas (TCGA) sequencing data (Figure S1d). These results suggested that the ERK5 pathway is activated by stress stimulation in the tumor microenvironment, and its ectopic activation is likely linked to lung cancer development.

### ERK5 promotes lung cancer cell proliferation, malignant transformation, and tumor xenograft growth

**Fig. 1:**
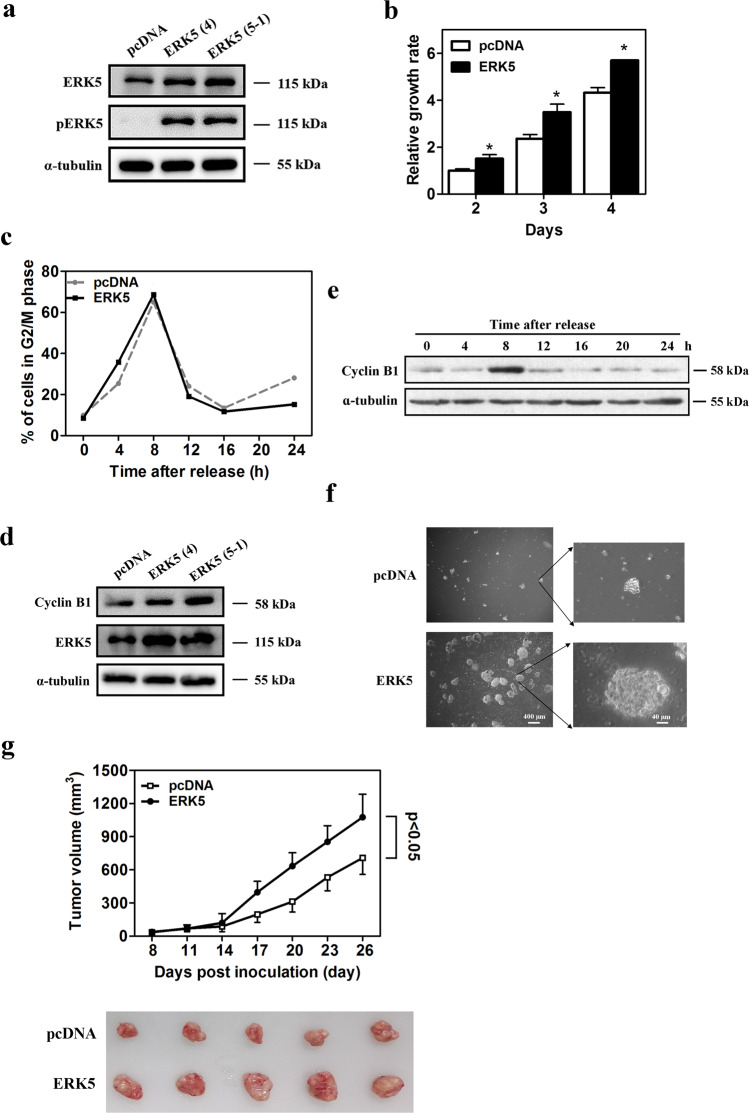
ERK5 promotes lung cancer cell proliferation and increased the malignant transformation ability of A549 cells. **a** The protein expression level of ERK5 and p-ERK5 in A549 cells stably transfected with either empty vector or vector expressing the ERK5 gene. **b** Stably transfected A549 cells were seeded 2000/well in 96-well plates, and cell growth was detected with MTT assays on days 2, 3, and 4. The data represent the mean ± SD; *p < 0.05 versus control. **c** Both ERK5-overexpression A549 cells and control cells were synchronized by double thymidine block and harvested at various time points after release. The cells were stained with PI, followed by flow cytometry analysis. **d** ERK5 overexpression and its effect on upregulation of cyclin B1 were detected via western blot, with ɑ-tubulin as the internal control. **e** ERK5-overexpression A549 cells were synchronized by double thymidine block and harvested at various time points after release. Then, the expression levels of cyclin B1 were detected via western blotting. **f** ERK5 overexpression and empty vector control A549 cells were cultured in soft agar and incubated for 3 weeks. The number and shape of colonies were examined. **g** The same number (2 × 10^6^) of A549 cells that stably expressed ERK5 and empty vector plasmids were injected subcutaneously into the right dorsum of each mouse; tumor growth rates were then compared. The data are presented as the mean ± SD, *p < 0.05. Representative images of excised tumors from each group are also shown (lower panel)

Although ERK5 plays a pivotal role in growth factor-induced cell proliferation, its role in cell cycle regulation is still unclear^34^. To investigate the role of ERK5 in lung cancer cell proliferation, we generated constructs allowing the expression of ERK5 that confer G418 resistance to A549 lung cancer cells. The overexpression of ERK5 in the stably transfected A549 cells was confirmed by western blotting (Fig. 1a). MTT assays indicated that ectopic expression of ERK5 significantly promoted A549 cell proliferation on days 2, 3, and 4 after detection (Fig. 1b). It has been reported that the stimulation of ERK5 activates nuclear factor κB (NF-κB) and plays a key role in the regulation of G2/M progression in HeLa cells^34^. Pro-proliferative effects are usually associated with the promotion of cell cycle progression. Therefore, the cell cycle distribution was determined using flow cytometric analysis. Both ERK5-overexpression and empty vector control A549 cells were treated with a double thymidine block. Through addition of serum, the synchronized G1/S phase A549 cells were then released for various periods of time up to 24 h. Following thymidine removal, the control A549 cells were distributed in G0/G1 (57.62%) and G2/M (9.91%) phases, while ERK5-overexpression cells were primarily distributed in G0/G1 (47.94%) phase, with 8.53% cells in G2/M phase. At 4 and 8 h after release from thymidine arrest, the ratio of ERK5-overexpression and control cells in G2/M phase was 35.78%:25.38% and 68.67%:65.04%, respectively. Moreover, as the cell cycle progressed from G2/M phase, the ratio of ERK5-overexpression cells in G2/M phase was lower than that of control cells (Fig. 1c). These results indicated that ERK5 could promote G2/M phase transition in A549 lung adenocarcinoma cells. The transition from G2 to M phase requires increased expression of cyclin B1, which is well known to be a key regulator of G2/M transition in both mitosis and meiosis^34^. As expected, western blotting results indicated that the protein level of cyclin B1 was obviously upregulated in ERK5-overexpression cells (Fig. 1d) and reached its peak value after release for 8 h (Fig. 1e). These data suggest that ERK5 can promote A549 lung cancer cell proliferation and G2/M cell cycle progression partly through upregulating cyclin B1 expression.

Because transformed cells have an ability to form anchorage-independent colonies in a semisolid medium, we next evaluated the action of ERK5 on the ability of A549 cells to form colonies. We found that ERK5-overexpression cells had greater capacity for anchorage-independent growth in soft agar. The colony number in soft agar increased from 24 per 60-mm plate in control cells to 58 per 60-mm plate in ERK5-overexpression cells. The colonies in the ERK5-overexpression group were also larger than those in the vector control group (Fig. 1f).

To further determine whether ERK5 affects the growth of tumor xenografts in mice, A549 lung cancer cells were transplanted into athymic nude mice. The same number (2 × 10^6^) of A549 cells that stably expressed ERK5 or empty vector plasmid were injected subcutaneously into the right dorsum of each mouse. We found that ERK5 overexpression significantly accelerated the growth of A549 solid tumors compared with the control group (on days 17–26, *p* < 0.05; Fig. 1g). These in vitro and in vivo experiments further support the notion that the malignant transformation ability and tumor xenograft growth were enhanced by ERK5 overexpression in A549 cells. These findings indicate that the ERK5 signaling pathway contributes to the malignant nature of lung cancer.

### ERK5 activity is stimulated by IR stress

**Fig. 2:**
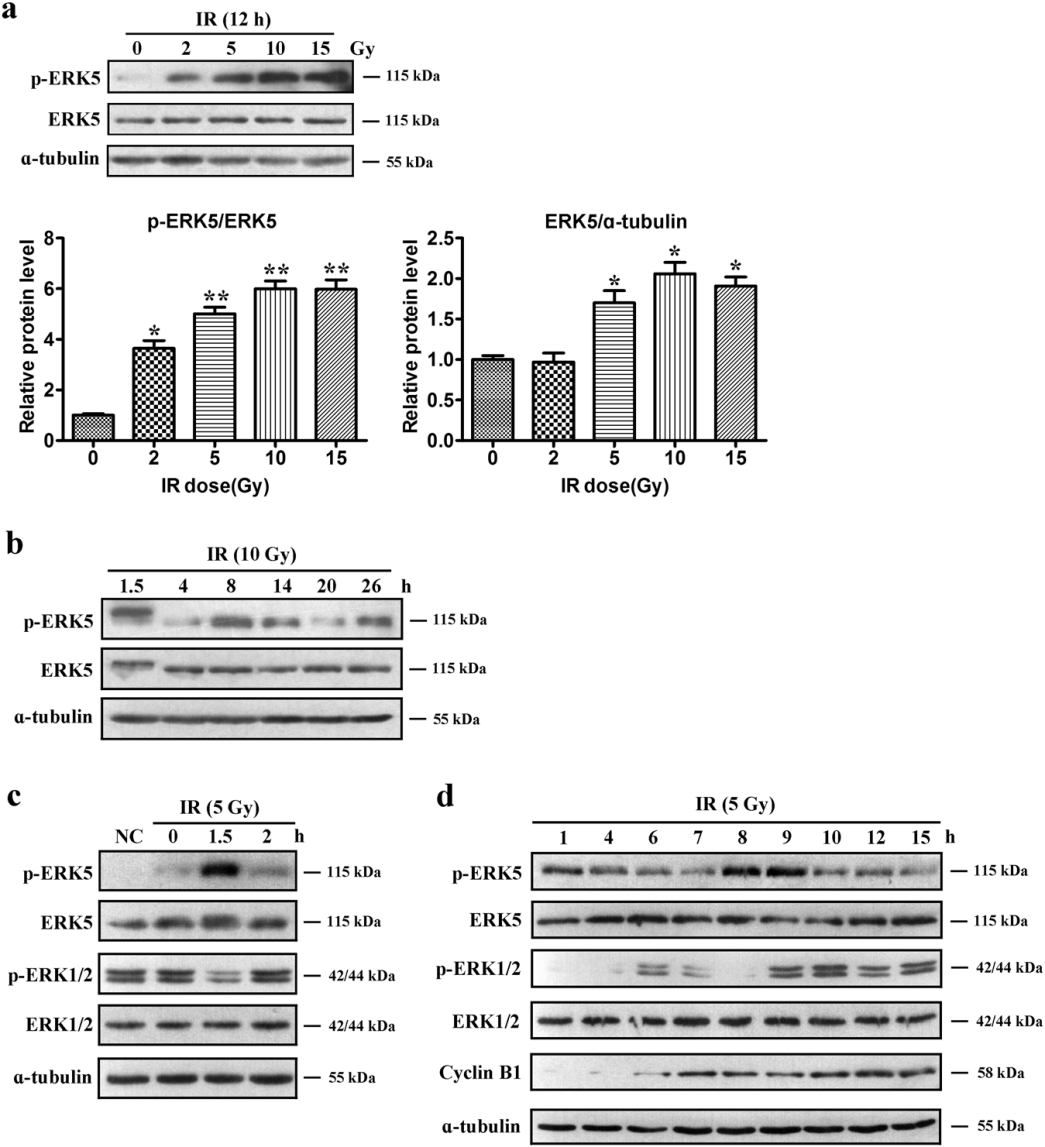
ERK5 expression is upregulated by IR stress. **a** A549 cells were exposed to different X-ray doses, and the expression levels of ERK5 and p-ERK5 were detected via western blotting at 12 h after exposure. Band intensity was quantified with ImageJ software. The results shown are representative of three different experiments. The data are presented as the mean ± SD; *p < 0.05 and **p < 0.01. **b** A549 cells were exposed to 10 Gy IR, and the expression levels of ERK5 and p-ERK5 were detected by western blotting at the indicated time after exposure. **c** A549 cells were exposed to 5 Gy IR, and the expression levels of ERK5, p-ERK5, ERK1/2, and p-ERK1/2 were detected by western blotting at 1.5 and 2 h after exposure. **d** A549 cells were exposed to 5 Gy IR, and the expression levels of ERK5, p-ERK5, ERK1/2, p-ERK1/2, and Cyclin B1 were detected by western blotting at the indicated time after exposure

Examination of the altered gene profiles of radioresistant cancer cells may provide new insights into the mechanisms underlying clinical radioresistance of tumors, including lung cancer^35^. ERK5 is characterized as a stress-sensitive MAPK. A variety of different environment stress stimuli, including fluid shear stress, osmotic stress, and oxidative stress, can active the ERK5 pathway, which contributes to the cell survival response. However, little is known about whether ERK5 functions are associated with radioresistance in cancer cells. We treated A549 cells with X-rays at different doses and determined ERK5 expression by western blotting after 12 h. The results indicated that ERK5 activity was significantly intensified in A549 cells upon exposure to IR stress (Fig. 2a). The phosphorylation level of ERK5 was considerably elevated after treatment with only 2 Gy, and the increase was dose-dependent. When cells were irradiated with a dose of 10 Gy, the level of ERK5 phosphorylation nearly reached its peak. The total protein level of ERK5 was also upregulated by 5 Gy or more; however, this change was not as obvious as the change in phosphorylation level (Fig. 2a). Next, we fixed the IR dose at 10 Gy and detected the expression of ERK5 at different recovery times. As shown in Fig. 2b, the phosphorylation level of ERK5 fluctuated periodically over time upon exposure to 10 Gy X-ray treatment. The change in the ERK5 phosphorylation level after 5 Gy X-ray treatment was similar to that with 10 Gy (Fig. 2c, d), indicating that ERK5 may play multiple roles in the response to IR. We also assessed the activity of ERK1/2 during IR. The results indicated that the response of ERK1/2 to IR stimulation was opposite that of ERK5, suggesting a compensatory regulation. The upregulation of ERK5 phosphorylation occurred at 1.5 and 8 h after IR treatment, while the phosphorylation of ERK1/2 was inhibited (Fig. 2c, d). In addition, cyclin B was found to be upregulated 7 h after IR treatment, at which time the ERK5 phosphorylation level was elevated again following X-ray treatment (Fig. 2d). In addition, a specific ERK5 inhibitor, XMD8-92, completely hindered the IR-induced phosphorylation of ERK5 in a concentration-dependent manner without affecting the phosphorylation of ERK1/2 at 12 h after IR treatment (Figure S2). These results suggest that ERK1/2 and ERK5 might act as interconnected signaling pathways in response to IR stimulation.

### Knockdown of ERK5 preferentially radiosensitizes NSCLC cells

**Fig. 3:**
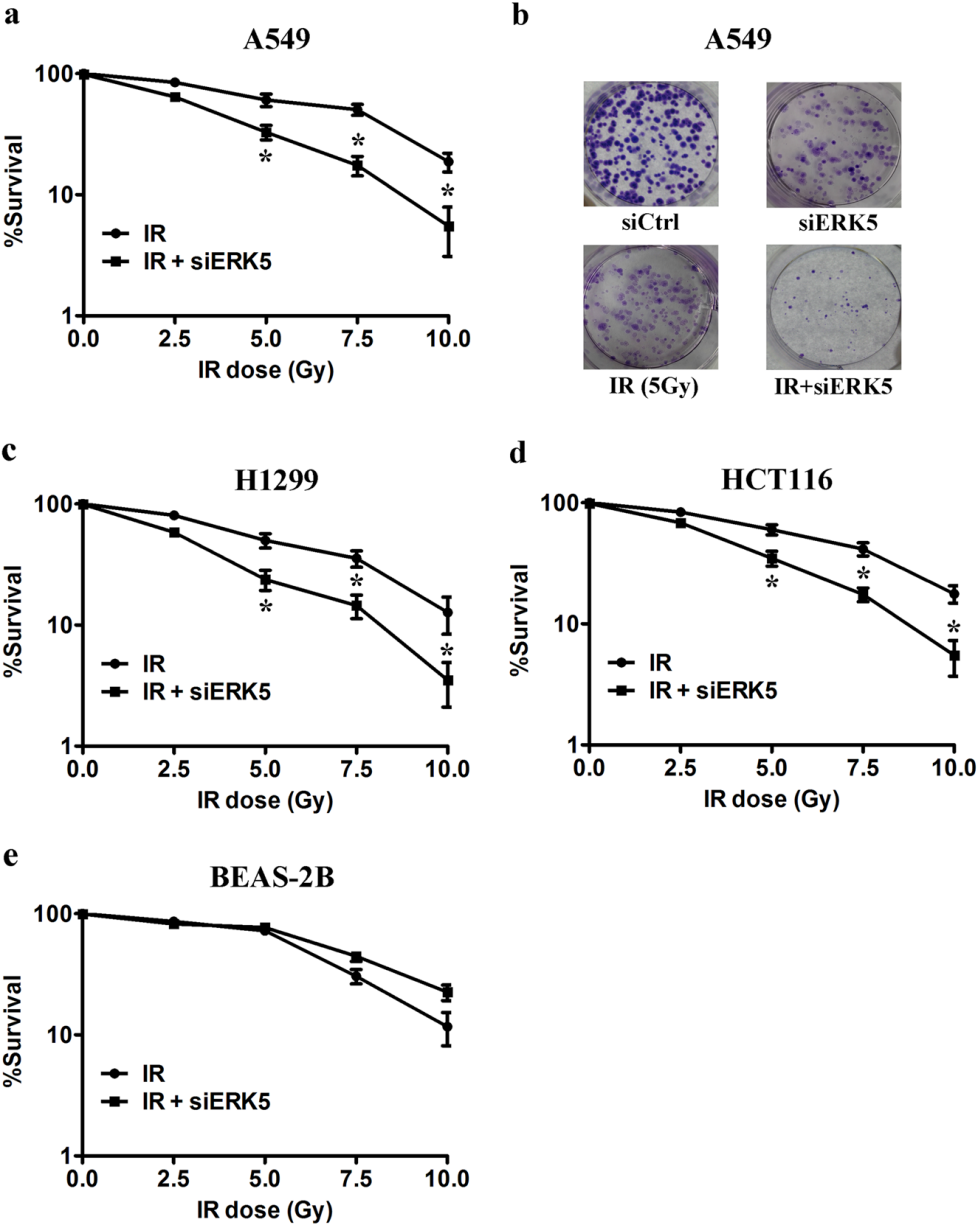
ERK5 knockdown radiosensitizes lung cancer cells. Cells were plated at 500 cells per well in a 6-well plate and after 24 h treated with the indicated dose of IR and/or ERK5 siRNA. Cells were then maintained for another 12 days. The formed colonies were fixed and stained. Colonies containing >50 cells were counted, and the colony formation percentage was determined for each cell line with respect to the nontreated controls. Survival curves for advanced human lung adenocarcinoma A549 cells (**a**) and representative images of stained colonies formed by A549 cells (**b**), H1299 cells (**c**), HCT116 cells (**d**), and transformed nonneoplastic human bronchial epithelial BEAS-2B cells (**e**). *p < 0.01 compared with the respective control

A549, H1299, HCT116, and BEAS-2B cells were transfected with siCtrl or siERK5, and ERK5 expression at 24 h after transfection was determined. Both the mRNA and protein levels of ERK5 were significantly decreased in these cells based on qPCR and western blot analyses (Figure S3). Two human NSCLC cell lines, A549 and H1299, were used to assess the effect of ERK5 on radioresistance using clonogenic survival assays. At a 5 Gy dose, there was a 39% decrease in A549 and a 50% decrease in H1299 colony formation, suggesting that H1299 cells may be more sensitive to IR than A549 cells (Fig. 3a–c). IR (2.5–10 Gy) inhibited colony formation by 16–81%, and inhibition increased to 35–94% when IR was combined with ERK5 knockdown in A549 cells (Fig. 3a). H1299 cells also displayed similar results (Fig. 3c). The DER at 50% suppression of colony formation was 2.03 in A549 and 1.51 in H1299 cells. In addition, knockdown of ERK5 increased the sensitivity of human HCT116 colon cancer cells to IR treatment, with a DER of 1.78 (Fig. 3d). Nevertheless, in nonneoplastic human bronchial epithelial cells (BEAS-2B), the same treatment with ERK5 knockdown did not sensitize cells to IR, and the treatment showed a DER of 0.89 at 50% suppression, indicating a radioprotective effect (Fig. 3e). We also found that the ERK5 inhibitor XMD8-92 could sensitize both A549 and H1299 cancer cells to IR treatment (Figure S4), which suggests a possible translation to the clinic.

### ERK5 protects NSCLC cells from IR-induced cell death

**Fig. 4:**
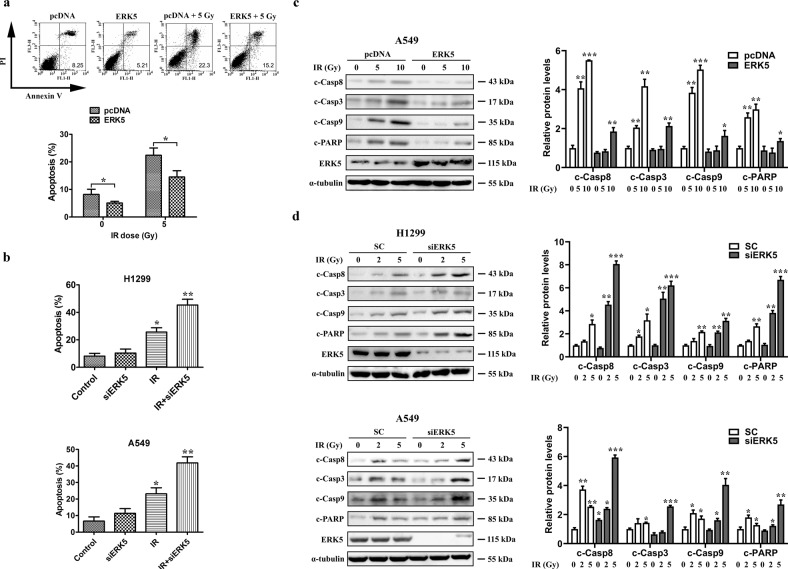
ERK5 inhibits IR-induced apoptosis in lung cancer cells. **a** A549 cells stably transfected with either empty vector or vector encoding ERK5 were treated with 5 Gy IR. After 16 h, all cells were harvested for flow cytometry analysis. Annexin V/PI-stained cells were analyzed, and the percentage of apoptotic cells was determined. The experiments were carried out independently in triplicate; representative data are shown. The data are presented as the mean ± SD. *p < 0.05. The Annexin V/PI double-staining profile of A549 cells is also included. **b** ERK5 was downregulated in H1299 and A549 cells through transfection with ERK5 siRNA. Cells were treated with IR, and cellular apoptosis was detected as in (**a**). **c** A549 cells stably transfected with either empty vector or vector encoding ERK5 were irradiated with IR at various doses and further cultured for 48 h. Whole-cell lysates were prepared and subjected to immunoblotting to detect cleaved caspase-8 (c-Casp8), cleaved caspase-3 (c-Casp3), cleaved caspase-9 (c-Casp9), and cleaved PARP (c-PARP) levels to reflect cellular apoptosis. ɑ-Tubulin levels were also detected as the loading control. **d** ERK5 was downregulated in H1299 and A549 cells after transfection with ERK5 siRNA. Cells were treated with IR, and cellular apoptosis was detected as in (**c**). The results shown are representative of three different experiments. Densitometric quantification of the immunoblot data in (**c**, **d**) is also shown. The data are presented as the mean ± SD; *p < 0.05, **p < 0.01, and ***p < 0.001

Given our finding that ERK5 reduces IR sensitivity in lung cancer cells, we next investigated the association between ERK5 and IR-mediated apoptotic cell death in NSCLC cells. We detected the apoptosis of A549 cells induced by 5 Gy X-ray irradiation using Annexin-V and PI double staining, followed by flow cytometry analysis 16 h after exposure. As shown in Fig. 4a, 5 Gy X-ray exposure significantly induced empty vector control A549 cell apoptosis (22.3%). However, ectopic expression of ERK5 effectively protected A549 cells from apoptosis after both sham and 5 Gy X-ray exposure, and the apoptotic rate was only 5.21% and 15.2%, respectively. On the other hand, we transfected H1299 cells with siERK5 or siCtrl and found an increase in apoptosis from 26% in cells treated with IR alone to 45% in cells treated with both IR and ERK5 knockdown (Fig. 4b). Similar results were obtained in A549 cells. Western blotting further showed that IR treatment at a low dose of 5 Gy was able to trigger apparent apoptosis in A549 cells, evidenced by observation of cleaved caspase-8, -3, -9, and cleaved PARP levels. However, a dose of at least 10 Gy was needed for IR to induce apoptosis when ERK5 was overexpressed in these cells, suggesting that ERK5 can protect A549 cells from IR-mediated apoptotic cell death (Fig. 4c). Additionally, we transfected H1299 and A549 cells with siERK5 or siCtrl and observed that knockdown of ERK5 promoted IR-mediated cell apoptosis, evidenced by the elevated cleaved caspase-8, -3, -9, and cleaved PARP levels (Fig. 4d). Moreover, the specific ERK5 inhibitor XMD8-92 also triggered apoptosis of A549 and H1299 cells receiving IR treatment, according to flow cytometry analysis (Figure S5). Overall, these results suggest that ERK5 can protect lung cancer cells from IR-mediated apoptosis, subsequently leading to inefficient cancer cell killing and radioresistance.

**Fig. 5:**
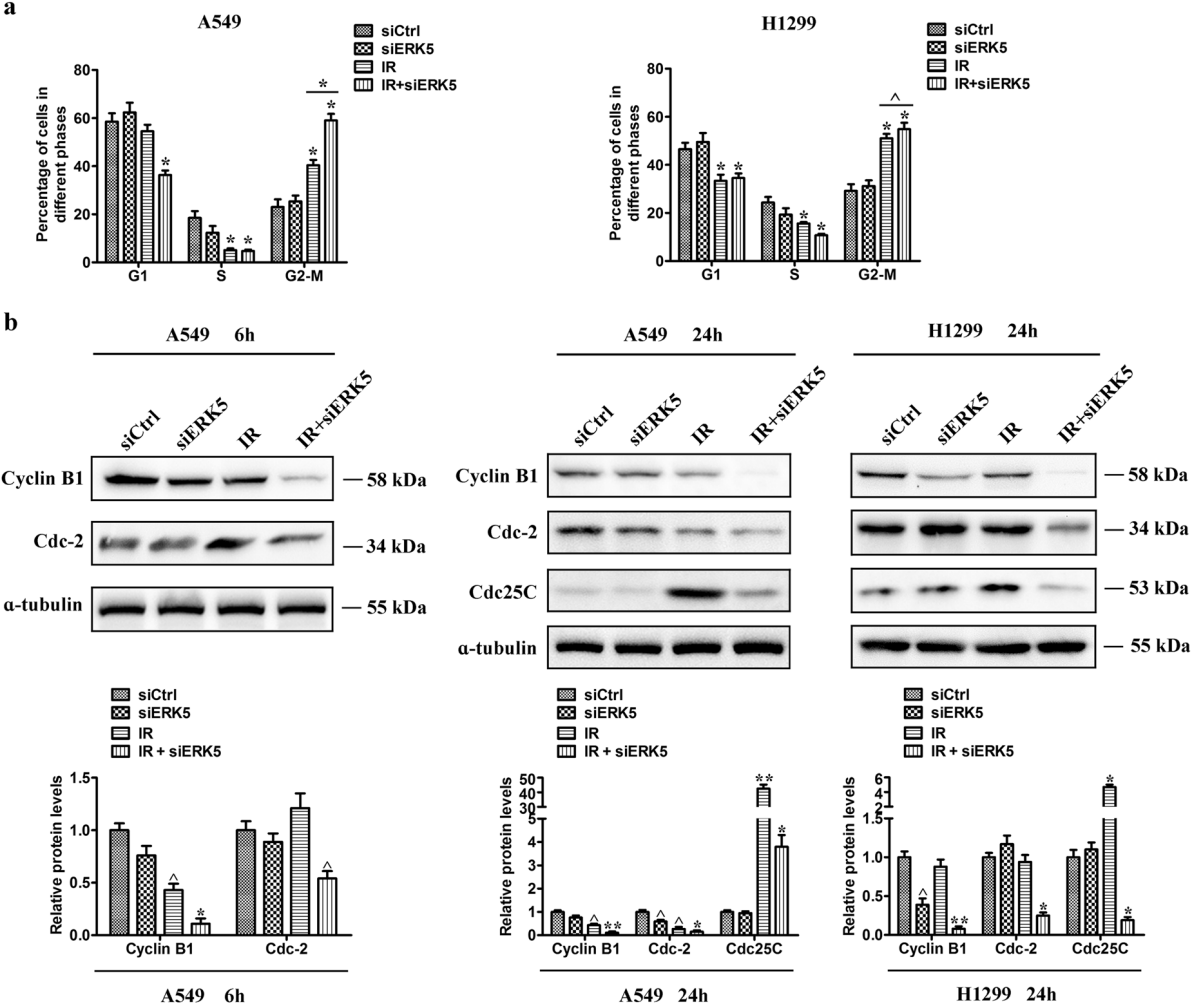
ERK5 knockdown potentiates IR-induced G2/M arrest. A549 and H1299 cells were exposed to IR with or without ERK5 siRNA treatment. After the treatment time points, cells were processed for cell cycle analysis using PI staining. **a** Quantitative data showing the cell cycle distribution in A549 (left) and H1299 cells (right) after treatment with IR (5 Gy) and/or ERK5 siRNA. *p < 0.01 and ^*p* < 0.05 compared with the respective control or indicated treatment. **b** Western blots for G2/M cell cycle-related proteins (Cyclin B1, Cdc2, and Cdc25C) at 6 and 24 h. Band intensity was quantified using ImageJ software. The results shown are representative of three different experiments. The data are presented as the mean ± SD. ^*p* < 0.05, *p < 0.01, and **p < 0.001

### ERK5 promotes the release of cell cycle arrest

Given that ERK5 protects NSCLC cells from IR-induced apoptosis, we wanted to investigate whether the expression level of ERK5 has an effect on cell cycle arrest in response to IR. We treated the ERK5-overexpression and empty vector control A549 cells with 5 Gy IR simultaneously. Then, the cells were collected at different time points, stained with PI, and examined for changes in cell cycle distribution using a flow cytometer. As shown in Figure S6a, irradiation of A549 cells with 5 Gy resulted in a delay in the progression into mitosis. In the first 0–24 h after irradiation, the empty vector control A549 cells showed G2/M arrest at approximately 6 h, with arrest reaching the highest level at 9 h. Approximately 22 h later, most cells were released from this arrest, and the G2 delay lasted for approximately 16 h. However, in the ERK5-overexpression A549 cells, G2/M arrest appeared at approximately 9 h after radiation and reached a peak value at 12 h. The cells exited from G2/M arrest approximately 20 h after exposure. The total G2 delay time for ERK5-overexpression A549 cells was approximately 11 h. In the next 24–48 h after radiation, G2/M arrest appeared again at approximately 33–41 h in the control cells, although the delay was not as long as in the first 0–24 h. In contrast, the ERK5-overexpression cells did not exhibit obvious G2/M delay. These results show that the overexpression of ERK5 resulted in a diminished G2 delay in irradiated A549 cells.

After exiting from G2/M arrest, more irradiated A549 cells transitioned into G1 phase. Many cells transitioned synchronously from G1 to S phase within 24 h. As shown in Figure S6b, the empty vector control group had a high percentage of cells in G1 phase after irradiation, which indicated that many cells were arrested in G1 phase after being irradiated, and the ratio of cells that re-entered S phase was decreased 24 h after exposure. In contrast, the percentage of cells that progressed from G1 to S phase was increased in ERK5-overexpression A549 cells compared with control group cells. A large number of ERK5-overexpression A549 cells entered S phase synchronously 24 h after exposure, and the number reached peaked at approximately 33 h. These results indicate that ERK5 overexpression in A549 cells promoted the release of cells from G1 arrest induced by irradiation and the transition from G1 to S phase.

To further determine the mechanisms associated with ERK5-induced radioresistance of NSCLC cells, we downregulated the expression of ERK5 in A549 and H1299 cells through transient transfection with ERK5 siRNA. Figure 5a shows the relative proportion of cells at each phase of the cell cycle (G0/G1, S, and G2/M). IR monotherapy increased the percentage of G2/M cells from 23% to 40% in the control group at 24 h, and the percentage was further elevated to 58.5% when IR was combined with ERK5 siRNA. A similar trend in cell cycle effects was observed in H1299 cells with combination treatment, although the effect was not as significant as that in A549 cells. Meanwhile, the expression of Cdc2 and Cyclin B1 decreased at 6 h after receiving the combined treatment. This effect lasted for up to 24 h, at which time we still detected IR-mediated elevation of Cdc25C, which was significantly decreased after treatment with IR and ERK5 siRNA, indicating prolonged G2/M arrest in the combined treatment group (Fig. 5b).

### ERK5 promotes IR-induced DNA damage responses

**Fig. 6:**
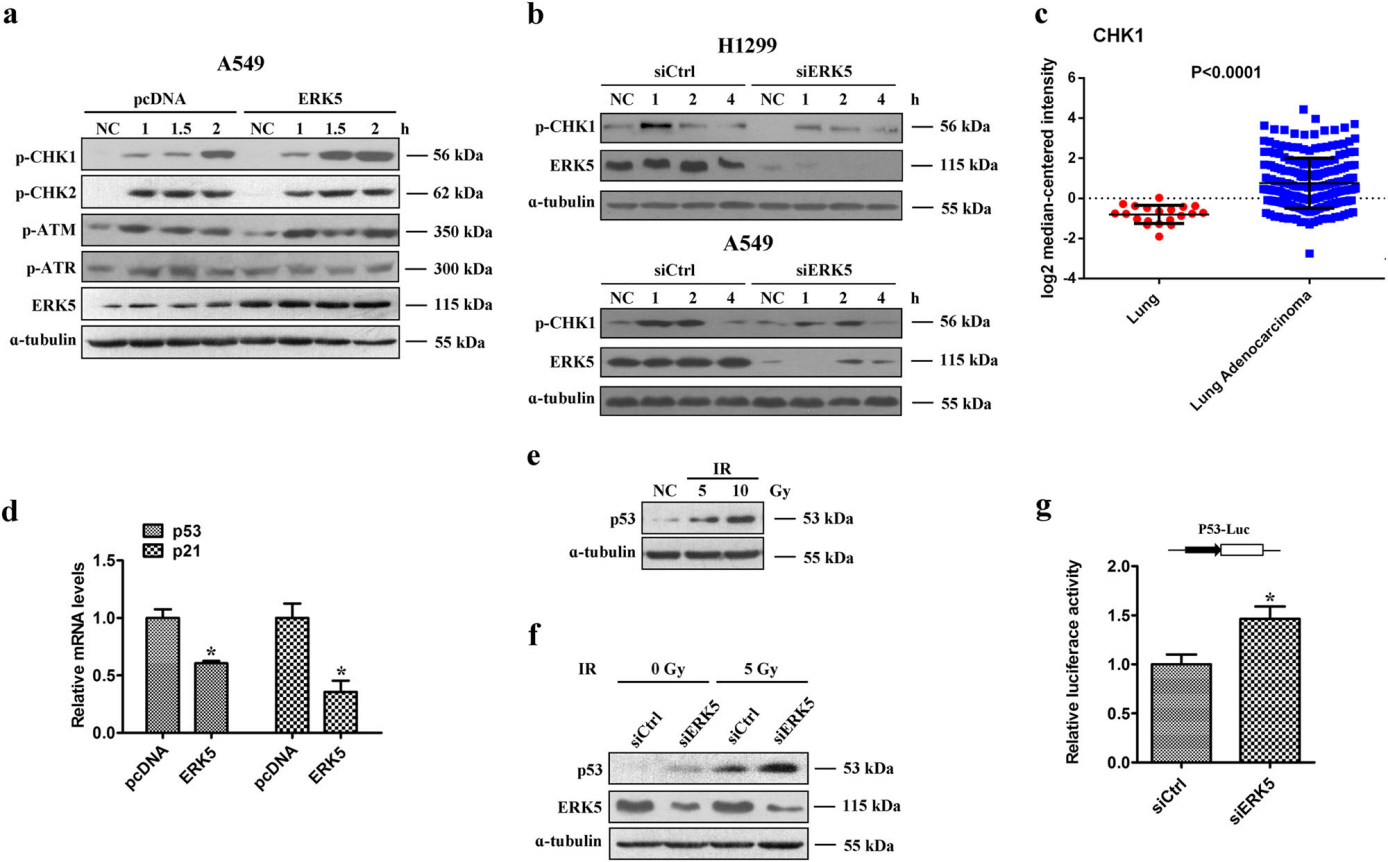
ERK5 promotes IR-induced phosphorylation of ChK1 in lung cancer cells. **a** A549 cells stably transfected with either empty vector or vector expressing ERK5 were treated with IR and further cultured for various time periods. Whole-cell lysates were prepared and subjected to immunoblotting to detect the levels of various checkpoint proteins. **b** ERK5 was downregulated in H1299 and A549 cells via transfection with ERK5 siRNA. Cells were treated with IR and further cultured for various time periods. Whole-cell lysates were prepared and subjected to immunoblotting to detect phosphorylated Chk1. ɑ-Tubulin was also detected as the loading control. **c** The expression levels of ERK5 in 226 lung adenocarcinoma tumor tissues and 20 normal tissues were obtained from TCGA. **d** A549 cells stably transfected with either empty vector or vector encoding ERK5 were harvested for RNA extraction and quantitative real-time PCR analysis using primers specific for human p53, p21, and GAPDH (internal control). The data represent the mean values of triplicate samples. *p < 0.05. **e** A549 cells were irradiated with IR at various doses, and the protein expression level of p53 was detected via western blotting. **f** A549 cells were transfected with control vector or ERK5 siRNA and treated with or without IR, and the protein expression level of p53 was detected via western blotting. **g** A549 cells were transfected with control vector or ERK5 siRNA. Transfected cells were then cotransfected with P53-Luc plasmid and pRL-TK Renilla vector. After 24 h, luciferase expression was measured. p53 luciferase activity was normalized to that of Renilla luciferase activity, and the results are expressed relative to the control values. The results are presented as the means ± SD from at least three independent experiments. *p < 0.05 versus control

Preferential activation of the DNA damage checkpoint response and an elevation in DNA repair capacity can trigger cancer cells to survive DNA-damaging therapy. We sought to clarify the mechanism by which ERK5 can prevent cells from apoptosis induced by IR. Thus, the effect of ERK5 overexpression on the activation of various DNA damage response proteins was determined. As shown in Fig. 6a, the overexpression of ERK5 in A549 cells resulted in an increased level of phosphorylated Chk1 upon IR treatment, without apparent influence on Chk2, ATM, and ATR phosphorylation. Next, we transfected H1299 and A549 cells with ERK5 siRNA to decrease the expression of ERK5 and observed a reduction in the phosphorylation level of Chk1 upon IR treatment in both cell lines (Fig. 6b). Overall, our results suggest that ERK5 can facilitate DNA damage checkpoint signaling by promoting Chk1 phosphorylation in response to IR. Furthermore, the level of Chk1 was found to be significantly higher in lung adenocarcinoma tissues than in normal lung tissues (Fig. 6c; *p* < 0.001). Kaplan–Meier survival analysis of lung cancer patients showed that cases with higher expression of ERK5 or Chk1 exhibited poorer overall survival (Figure S7). These data suggest that ERK5 and Chk1 may serve as indicators for lung adenocarcinoma diagnosis and prognosis.

### ERK5 inhibits the p53 pathway

The tumor suppressor p53 is critical for the DNA damage-induced checkpoint, which coordinates cell cycle progression with DNA repair. Whether ERK5 participates in cancer progression through the p53 pathway remains unknown. Thus, we detected the direct impact of ERK5 on the expression of p53/p21 using loss-of-function and gain-of-function strategies in the p53 wild-type cell line A549. We found that the mRNA levels of p53 and p21 were significantly downregulated in ERK5-overexpression cells (Fig. 6d). In addition, we demonstrated that IR could lead to an increase in the p53 protein level in a dose-dependent manner (Fig. 6e), and we detected a dramatic increase in the p53 protein level following ERK5 knockdown combined with IR (Fig. 6f). We also examined p53 promoter activity. Downregulation of ERK5 by siRNA significantly increased p53 activity (Fig. 6g).

### ERK5 enhances homologous recombination repair upon IR treatment

**Fig. 7:**
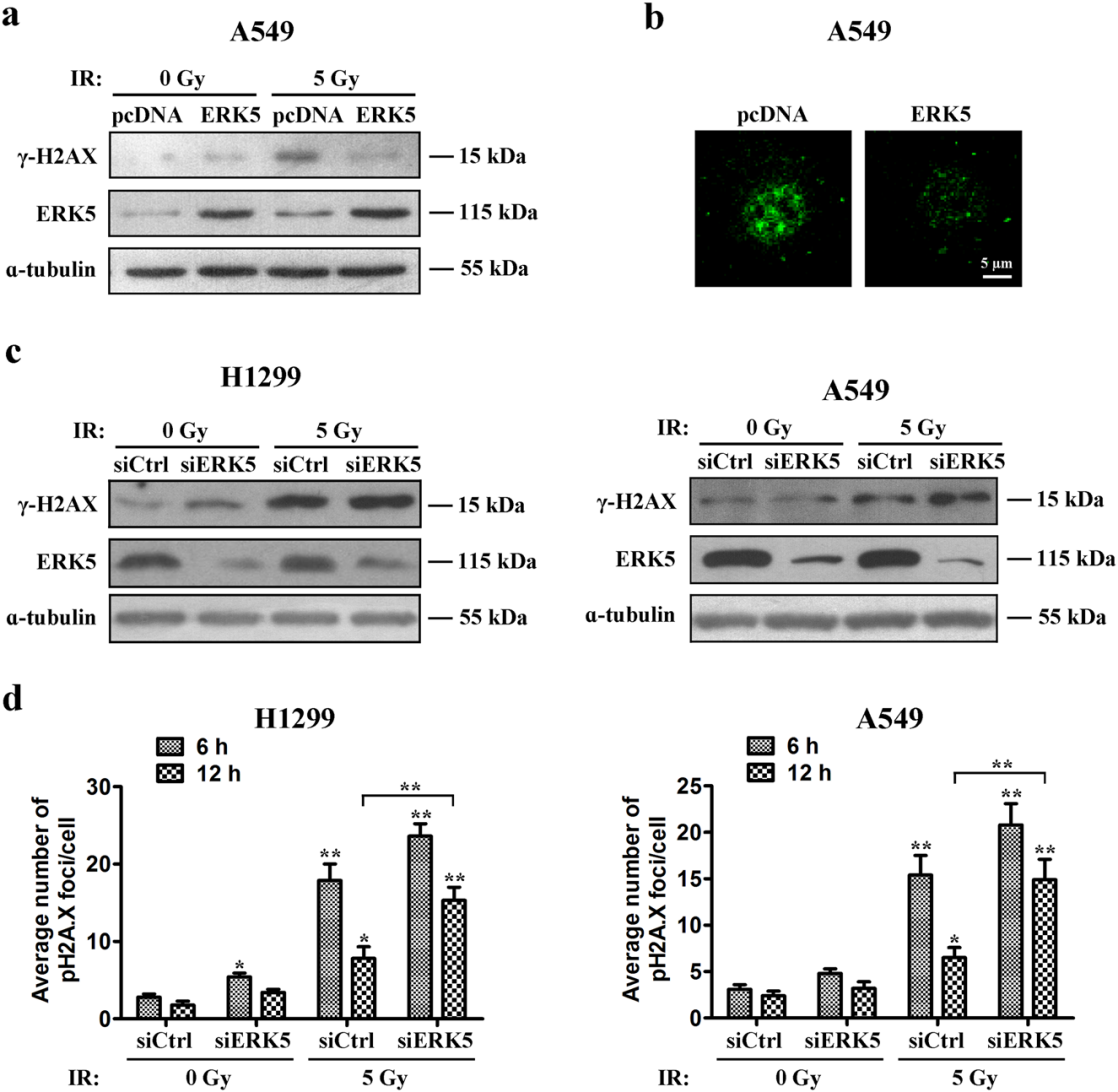
ERK5 facilitates homologous recombination to repair IR-induced DSBs. **a**, **b** A549 cells stably transfected with either empty vector or vector expressing ERK5 were treated with 5 Gy IR and further cultured for 6 h. Whole-cell lysates were prepared and subjected to immunoblotting to detect ERK5 and phosphorylated H2AX (γH2AX) levels. ɑ-Tubulin levels were also detected as the loading control (**a**). Immunofluorescent staining for γH2AX was performed on control and ERK5 overexpression A549 cells fixed at 6 h following 5 Gy X-ray irradiation treatment (**b**). **c**, **d** H1299 and A549 cells were transfected with control vector or ERK5 siRNA, treated with or without IR, and further cultured for 6 h. Whole-cell lysates were prepared and subjected to immunoblotting to detect ERK5 and phosphorylated H2AX (γH2AX) levels. ɑ-Tubulin was also detected as the loading control (**c**). Quantitation of the number of pH2A.X foci in H1299 and A549 cells after 6 and 12 h of treatments are also shown (**d**). *p < 0.05 and **p < 0.01 compared with the respective control or indicated treatment

IR-induced apoptosis is usually associated with increased DNA damage and/or impaired DNA repair systems^36^. Based on these findings, we hypothesized that one of the potential biological functions of ERK5 is related to DNA damage repair. It is well known that the morphology and number of γ-H2AX foci undergo substantial dynamic changes during the DNA damage response^37^. IR induces a large variety of DNA lesions, including single- and double-strand breaks (DSBs) and base and sugar damage, among them, DSBs are the most dangerous. Thus, DSBs are widely considered to be the typical form of IR-induced DNA damage. Double-strand DNA damage can be represented by phosphorylation of a core histone variant, histone H2AX. After irradiation with IR, H2AX is rapidly phosphorylated. The γ-H2AX foci continue to grow for approximately 1 h and then disappear slowly over time, and there is always a constant number or percentage of γ-H2AX formed per DSB. After DNA is repaired, γ-H2AX is dephosphorylated. The persistence of γ-H2AX after IR is particularly significant because it can be used as a marker of radiosensitivity. We treated A549 cells with 5 Gy X-ray irradiation and harvested the cells 6 h after exposure. The expression level of γ-H2AX was detected by western blot. We found that a high level of H2AX phosphorylation was maintained in control cells 6 h after exposure, while in ERK5-overexpression A549 cells, the intensity of the γ-H2AX signal was reduced to a low level (Fig. 7a). Then, the morphology of γ-H2AX foci was observed. As expected, large γ-H2AX foci were formed in empty vector control A549 lung cancer cells 8 h after exposure (Fig. 7b, left panel). In contrast, this morphological progression was not observed in ERK5-overexpressing A549 cells despite the initial formation of small γ-H2AX speckles as observed in the control (Fig. 7b, right panel). The number and intensity of the γ-H2AX foci were much less than those in the control. Conversely, more γ-H2AX foci were observed in H1299 and A549 cells transfected with ERK5 siRNA than in cells transfected with control siRNA at 6 h post-IR (Fig. 7c). Additionally, we found that in H1299 cells the number of pH2AX foci was increased 6 h after IR exposure and was reduced by 56% at 12 h, while in the presence of ERK5 siRNA, the number was elevated by 32% (Fig. 7d, left). Moreover, at 12 h after IR treatment, the number of pH2AX foci in the presence of ERK5 siRNA was about two-fold that in cells treated with IR alone (Fig. 7d, left), indicating that downregulation of ERK5 halted the repair of IR-induced DSBs. Similar results were observed in A549 cells (Fig. 7d, right). These data suggest that ERK5 has a critical role in the IR-induced DNA damage response, and ERK5 downregulation-mediated radiosensitization involves inhibition of repair of IR-induced DNA damage.

### ERK5 knockdown inhibits LLC lung cancer growth in vivo

**Fig. 8:**
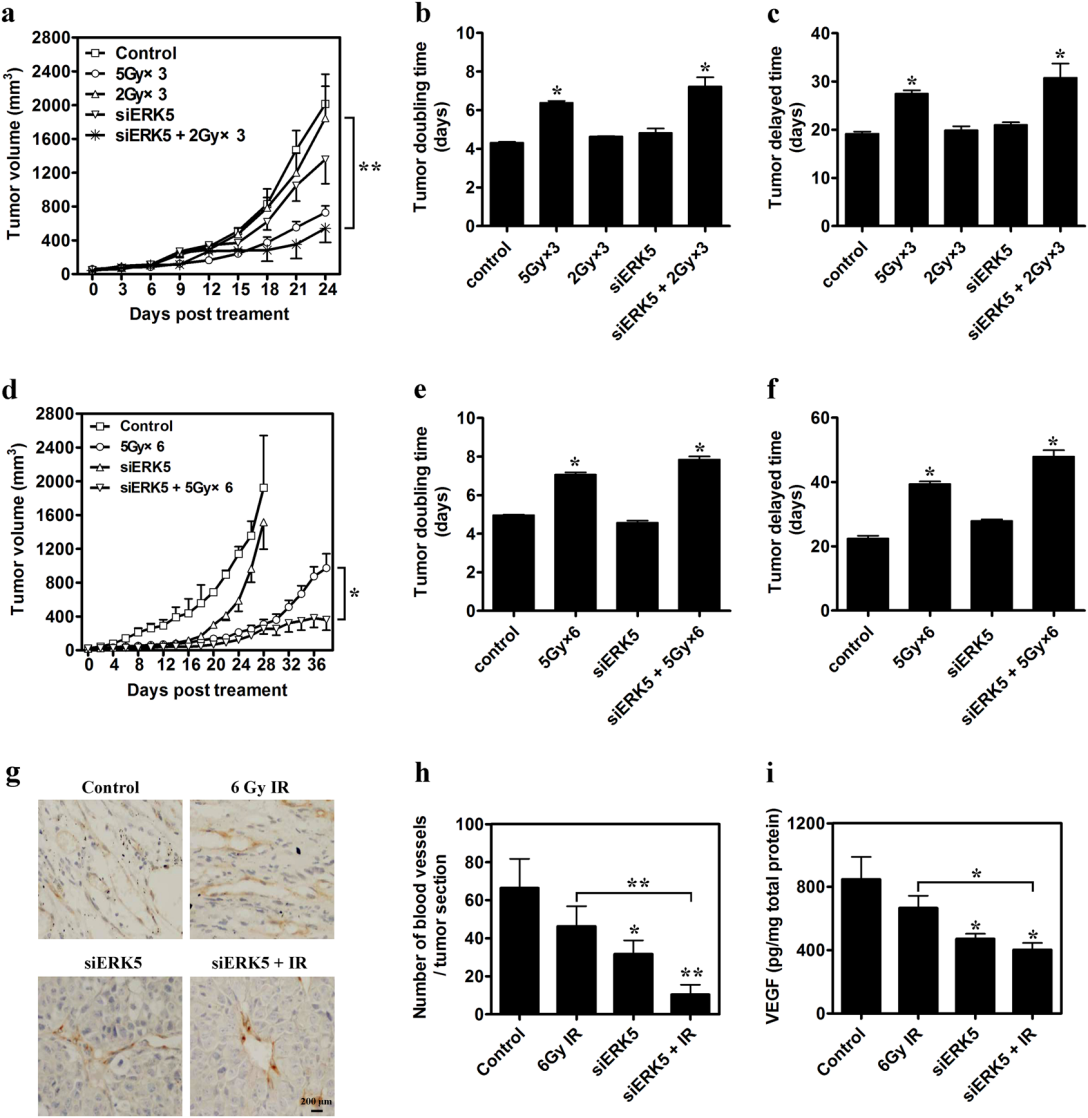
ERK5 knockdown inhibits LLC tumor growth and increases tumor radiosensitivity. **a**–**c** Tumor growth was suppressed in LLC-bearing C57BL/6J mice treated with ERK5 knockdown combined with low-dose IR. When visible tumors were formed, local irradiation treatments (cumulative dose of 6 Gy) were fractionally administered on days 0, 2, and 4, and constructs expressing either Luc shRNA or ERK5 shRNA were injected into the tumor mass on days 1, 3, and 5. The tumor growth inhibitory effects of different treatments were compared (**a**). Tumor doubling time (**b**) and tumor delay time (**c**) are also shown. **d**–**f** Tumor growth was suppressed in LLC-bearing C57BL/6J mice treated with ERK5 knockdown combined with high-dose IR. When visible tumors were formed, local irradiation (total dose of 30 Gy) was fractionally administered on days 0, 2, 4, 6, 8, and 10, and a construct expressing either Luc shRNA or ERK5 shRNA was injected into the tumor mass on days 1, 3, and 5. The tumor growth inhibitory effects of different treatments were compared (**d**). Tumor doubling time (**e**) and tumor delay time (**f**) are also shown. **g**, **h** Blood vessel density within tumors was characterized by anti-CD31 immunostaining using an anti-mouse CD31 monoclonal antibody (**g**) and determined by the average number of vessels in 3 regions of the highest density at ×200 magnification in each section (the assay was repeated in 4 sections per mouse, and 3 mice were tested) (**h**). **i** Total protein was extracted from LLC tumors, and the intracellular VEGF level was detected via ELISA using 100 μg total protein per well. The data are presented as the mean ± SD; *p < 0.05 and **p < 0.01 compared with the respective control or indicated treatment

To determine whether ERK5 affects the growth of established tumors in mice, LLC lung cancer cells were transplanted into C57BL/6J mice. Three distinct shRNAs that specifically target ERK5 were designed and constructed and then used to transfect LLC cells, and the efficiency of target gene knockdown was determined by RT-PCR and immunoblotting. The results indicate that ERK5 shRNA-2 substantially reduced ERK5 expression (Figure S8). For in vivo animal experiments, when visible tumors were formed, local IR with 6 Gy (2 Gy, 3 times) was fractionally administered on days 0, 2, and 4, and a construct encoding either siCtrl (Luc shRNA) or siERK5 (ERK5 shRNA-2) was injected into the tumor mass on days 1, 3, and 5. As shown in Fig. 8a, 6 Gy IR treatment did not effectively inhibit LLC lung cancer growth, and the tumor volume on day 24 reached 1846.70 mm^3^, which was close to the tumor size in the control group (2012.80 mm^3^). However, ERK5 knockdown obviously reduced LLC tumor growth and significantly increased the sensitivity of RT at the dose of 6 Gy. On day 24, the size of tumors treated with siERK5 was only 1358.12 mm^3^, which was 67.48% the size of tumors in the control group. ERK5 siRNA combined with 6 Gy IR displayed a powerful anti-tumor effect. On day 24, the tumor volume was only 541.67 mm^3^, which was 29.33% and 39.88% the size of tumors in mice receiving 6 Gy RT or ERK5 siRNA alone, respectively (Fig. 8a, *p* < 0.05). The combined therapy exerted a more obvious effect on tumor suppression compared with high-dose RT at 15 Gy fractionally administered (5 Gy, 3 times) (729.36 mm^3^). The tumor doubling time (7.01 days) and tumor delay time (29.64 days) in the siERK5 combined with 6 Gy RT group were also obviously increased (*p* < 0.05) compared with those in the control group and 6 Gy IR treatment group (Fig. 8b, c, Table 1).

**Table 1:** Regression analysis of treatment effects of low-dose IR combined with ERK5 knockdown on tumor growth

Treatment	n	Growth curve *v* (days)	r	Tumor doubling time (days)	Tumor growth delay (days)
**Control**	8	ln(v) = 0.1609 d + 3.833	0.9955	4.31 (4.24–4.40)	19.11 (18.40–19.96)
**5** **Gy** × **3**	8	ln(v) = 0.1083 d + 3.937	0.9844	6.40 (6.21–6.54)	27.43 (26.26–28.69)
**2** **Gy** × **3**	8	ln(v) = 0.1494 d + 3.957	0.9905	4.64 (4.59–4.68)	19.75 (18.49–21.36)
**ERK5 RNAi**	8	ln(v) = 0.1422 d + 3.920	0.9736	4.88 (4.39–5.19)	21.01 (19.98–21.95)
**ERK5 RNAi** **+** **2** **Gy** × **3**	8	ln(v) = 0.0989 d + 3.976	0.9356	7.01 (6.53–8.14)	29.64 (26.26–36.38)

To further understand whether ERK5 knockdown improves lung cancer cell sensitivity to exposure at high IR doses, the generated lung cancer xenografts were fractionally administered high-dose IR (30 Gy) and/or ERK5 knockdown. As shown in Fig. 8d, 30 Gy RT (5 Gy, 6 times, on days 0, 2, 4, 6, 8, and 10), siERK5 (on days 1, 3, 5, 7, 9, and 11) alone, or siERK5 plus RT significantly inhibited tumor growth until day 18. However, ERK5 knockdown alone did not efficiently inhibit tumor growth after day 18, while the tumor growth was slower in the two groups receiving either 30 Gy RT alone or the RT combined with siERK5 therapy. The combined therapy suppressed lung cancer growth more significantly. On day 38, the tumor volume was only 360.14 mm^3^, which was 37.04% that in the group receiving 30 Gy RT alone (*p* < 0.05). The tumor doubling time (7.88 days) and tumor delay time (47.52 days) induced by siERK5 combined with 30 Gy RT were also obviously increased (*p* < 0.05) compared with the control group and 30 Gy RT group (Fig. 8e, f, Table 2). These findings suggest that ERK5 is one of the important pathways related to the generation of cancer cell radioresistance and targeted disruption of ERK5 signaling could improve the anti-tumor effect of RT.

**Table 2:** Regression analysis of treatment effects of high-dose IR combined with ERK5 knockdown on tumor growth

Treatment	n	Growth curve *v* (days)	r	Tumor doubling time (days)	Tumor growth delay (days)
**Control**	8	ln(v) = 0.1399 d + 3.793	0.9485	4.96 (4.91–5.02)	22.26 (21.00–23.96)
**5** **Gy** × **6**	8	ln(v) = 0.0978 d + 3.065	0.9902	7.09 (6.86–7.25)	39.29 (37.87–40.84)
**ERK5 RNAi**	8	ln(v) = 0.1508 d + 2.706	0.9706	4.60 (4.38–4.74)	27.86 (26.96–28.81)
**ERK5 RNAi** **+** **5** **Gy** × **6**	8	ln(v) = 0.088 d + 2.726	0.9588	7.88 (7.56–8.11)	47.52 (44.77–51.51)

### ERK5 knockdown inhibits LLC tumor neovascularization

Embryos deficient in the ERK5 gene exhibit angiogenic failure and cardiovascular defects^15^. In ERK5 flox/flox mice, induced deletion of host ERK5 strongly inhibits the growth of B16F10 and LLC tumor xenografts and is associated with a significant decrease in vascular density^16^. However, it is unclear whether targeted disruption of ERK5 in lung cancer cells can inhibit tumor neovascularization. We first examined the effect of ERK5 knockdown on tumor vascular density in LLC solid tumors. When the LLC tumor volume reached approximately 50 mm^3^, the tumor was administered 6 Gy (2 Gy, 3 times, on days 0, 2, and 4), siERK5, or treated with both RT and siERK5 in combination. On the 1st day post-treatment, an anti-CD31 antibody was used to determine blood vessel density in tissue sections from LLC tumors, followed by standard IHC. The results indicated that the tumor vessel density was dramatically reduced after exposure to siERK5 alone, compared with that in control tumors. Treatment with 6 Gy IR combined with siERK5 reduced tumor neovascularization more clearly, with a 15.6% decrease in vascularization compared with that in the control group (Fig. 8g, h). We further assessed whether the ERK5 knockdown-induced reduction in vessel density was associated with VEGF expression in lung cancer. ELISA results showed that the expression of VEGF was significantly inhibited by siERK5 alone or in combination with local irradiation (Fig. 8i), which was well correlated with the decrease in vessel density. These results imply that targeted disruption of ERK5 in cancer cells, such as lung cancer cells, reduces VEGF expression, tumor angiogenesis, and tumor growth.

### ERK5 knockdown enhances radiation-induced A549 tumor growth inhibition

**Fig. 9:**
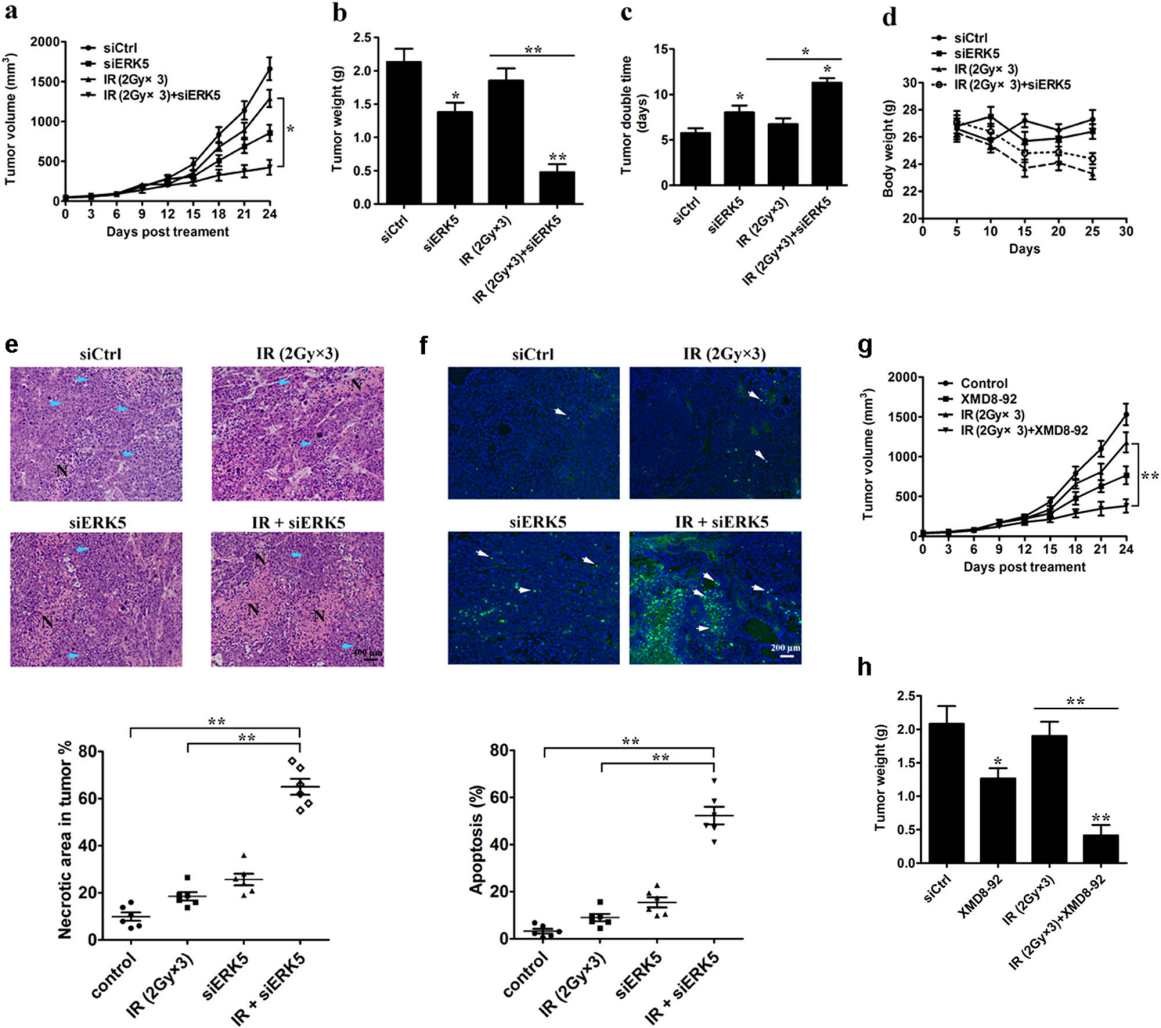
ERK5 knockdown enhances radiation-induced tumor growth inhibition of human lung cancer A549 xenografts in athymic nude mice. Mice were given a s.c. injection of A549 cells (2 × 10^6^) and monitored for tumor growth until the tumor size reached approximately 50 mm^3^. Then, local irradiation with 6 Gy was fractionally administered on days 0, 2, and 4, and a construct encoding either siCtrl or siERK5 was injected into the tumor mass on days 1, 3, and 5. **a** The tumor growth inhibitory effects of different treatments were compared. **b** Tumor weight/mouse at the end of the study. Tumor doubling time (**c**) and mean body weight per mouse (**d**) are also shown. **e** Determination of tumor necrosis after combined treatment with ERK5 siRNA and IR. Tumor necrosis areas are shown by H&E staining and were observed under a light microscope (×100). The viable tumor cells are indicated by a blue arrow. Tumor necrosis was determined with ImageJ software. Two sections/mouse from three mice were prepared. **f** Determination of tumor apoptosis after combined treatment with ERK5 siRNA and IR. TUNEL assays were used to detect apoptotic cells (original magnification, ×200). Cells positive for TUNEL staining are indicated by a white arrow. The ratio of apoptotic cells to total cells: TUNEL-positive cells were counted in three fields with the highest density of positively stained cells in each section to determine the percentage of apoptotic cells. **g**, **h** Mice were given a s.c. injection of A549 cells (2 × 10^6^) and monitored for tumor growth until the tumor size reached approximately 50 mm^3^. Then, mice were treated with local irradiation (6 Gy, fractionally administered on days 0, 2, and 4), XMD8-92 (25 mg/kg) or a combination of the treatments. The tumor growth inhibitory effects of different treatments were compared (**g**). Tumor weight/mouse at the end of the study is shown (**h**). The data are presented as the mean ± SD, *p < 0.05 and **p < 0.01 compared with the respective control or indicated treatment

Given that the knockdown of ERK5 was able to sensitize LLC lung cancer cells to IR treatment, we further validated this effect using an A549 xenograft model. As described in Materials and methods, when tumors reached an average volume of approximately 60 mm^3^, tumor-bearing mice were randomly divided into 4 groups and administered IR and/or siERK5. IR and siERK5 suppressed the growth of tumors by 21% and 49% compared with that of control tumors, respectively; however, their combination led to 75% growth inhibition compared with the control (Fig. 9a). Similarly, tumor weight was decreased by 36%, 14%, and 71% in the siERK5, IR, and combination IR and siERK5 groups compared with the control group, respectively (Fig. 9b). The tumor weight and volume were reduced by 74% and 68% by the combined treatment compared with IR monotherapy, respectively. Tumor doubling time was prolonged from 5.76 days in the siCtrl-treated group, 8.03 days in the siERK5-treated group, and 6.73 days in the IR-treated group to 11.35 days in the group treated with IR and siERK5 in combination (CI = 1.72; Fig. 9c), suggesting a synergistic effect of IR and siERK5. Additionally, IR monotherapy led to a 14.6% decrease in body weight at the end of treatment; however, siERK5 treatment reversed this loss by 4.7% (Fig. 9d), indicating that siERK5 treatment partially showed a radioprotective response in mice.

Light microscopy further revealed that tumor tissues in mice that received siERK5 plus IR therapy showed more severe necrosis relative to control, siERK5 or IR monotherapy (Fig. 9e). The percentage of necrotic area in tumors was elevated from 9.8% in the siCtrl-treated group, 18.5% in the IR-treated group, and 25.6% in the siERK5-treated group to 65.2% in the siERK5- and IR-treated group. The control, siERK5, and IR monotherapy groups showed tissue necrosis interspersed with viable tumor cells, while large areas of continuous necrosis were observed in tumors treated with siERK5 and IR in combination (Fig. 9e). TUNEL assays were also performed to clarify the effect of combined treatment on apoptotic cell death. We found that ERK5 knockdown significantly facilitated IR-induced apoptosis in the group that received the combined treatment (Fig. 9f).

Additionally, to examine the efficacy of IR and XMD8-92 in combination in vivo, we assessed the effects of single therapy or their combination in A549 xenografts. We found that, compared with control group, XMD8-92 (25 mg/kg) twice a day for 24 days resulted in a 50% tumor growth inhibition; however, combined treatment with XMD8-92 (25 mg/kg) and IR (6 Gy) produced a markedly greater anti-tumor effect than either monotherapy, paralleling data obtained in vitro (Fig. 9g, h). Taken together, these data indicate that the knockdown of ERK5 or pharmacological inhibition of ERK5 activity can improve the effect of IR therapy against lung cancer cell growth both in vitro and in vivo.

## Discussion

**Fig. 10:**
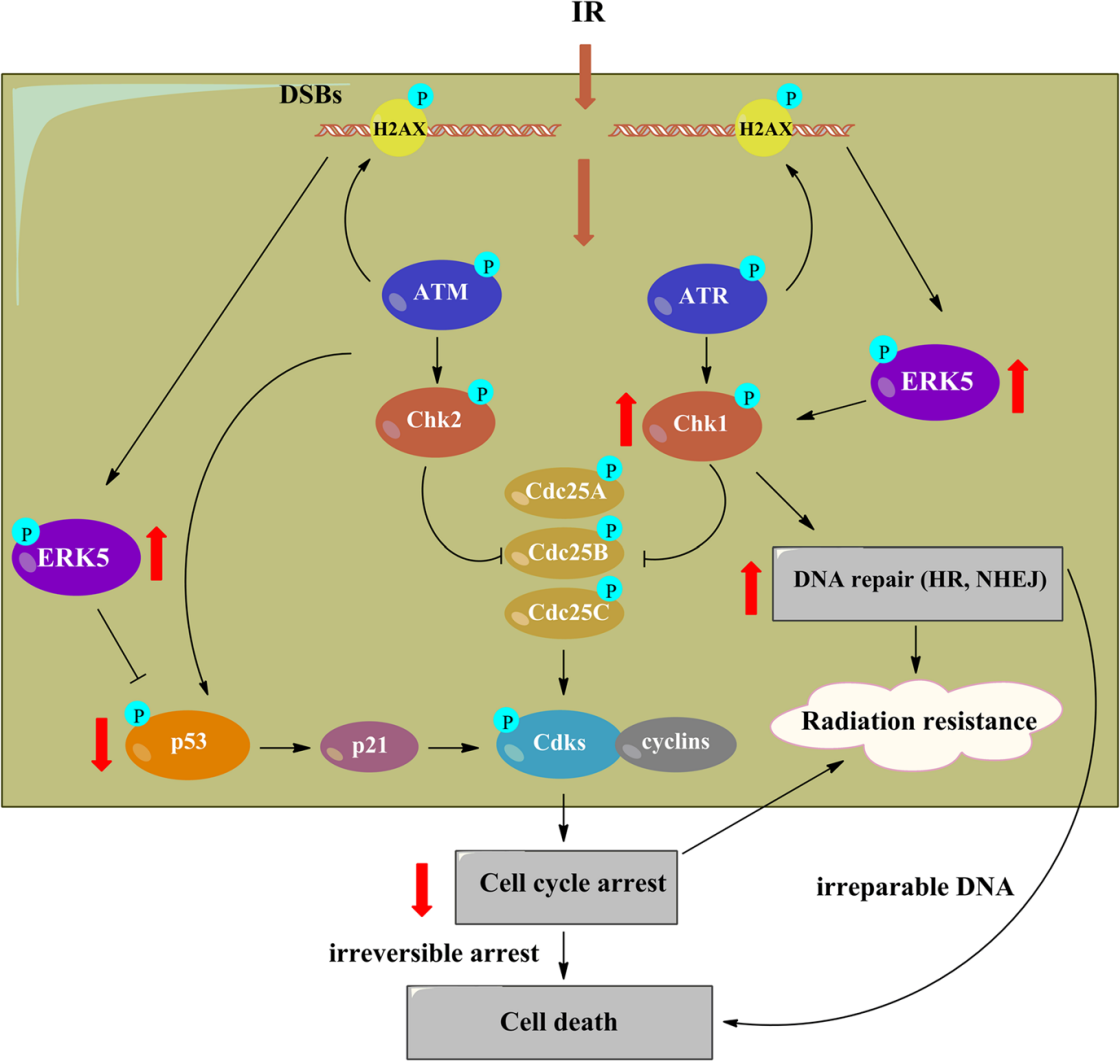
Schematic of the potential mechanisms by which ERK5 inhibits the effect of lung cancer cell radiotherapy

The current study showed for the first time that ERK5 might be critical for Chk1 activation in response to IR and that ERK5 promotes DNA repair, thereby enhancing the resistance of NSCLC cells to radiotherapy. We also demonstrated the radiosensitizing effects of ERK5 knockdown or pharmacological inhibition for lung cancer therapy. Downregulation of ERK5 sensitized NSCLC cells to radiotherapy by (1) enhancing and sustaining the IR-induced G2/M cell cycle arrest and (2) decreasing the ability of lung cancer cells to repair DNA damage induced by IR (Fig. 10). These mechanisms ultimately lead to decreased cell proliferation, reduction in clonogenicity, and elevated cell apoptosis, thus enhancing the radiotherapeutic response.

ERK5 has recently attracted more interest due to its critical role in cell survival^38^, which has been found to be related to cancer by analysis of ERK5 expression in human tumors^17^. Here, we show that ERK5 is significantly upregulated in solid lung tumor growth progression and potently promotes cell proliferation and cell cycle progression in A549 lung adenocarcinoma, at least in part by upregulating cyclin B1, which is an essential modulator in controlling G2/M phase transition. This mechanism is quite distinct from the ERK5 cascade, an important contributor to cyclin D1 regulation in several breast cancer cell lines, and cyclin D1 regulates the cell cycle G1 to S transition^39^. This finding indicates that ERK5 is a novel cell cycle modulator and essential for regulating multiple genes related to cell cycle phase control.

Among the four identified mammalian MAPK pathways (ERK1/2, ERK5, p38, and JNK), the p38 and JNK pathways primarily mediate cellular responses to stress stimuli, whereas ERK5 and ERK1/2 are largely involved in the regulation of intracellular signaling from growth factors^7^. Our results clearly indicate that ERK5 expression is upregulated by X-ray irradiation stress and that ERK5 overexpression effectively protects against lung cancer cell apoptosis induced by exposure to X-ray irradiation. Furthermore, this effect of ERK5 appears to be associated with an increased DNA damage repair response. In response to DNA damage, the kinases Chk1 and Chk2 are phosphorylated by the DNA damage sensors ATR and ATM, respectively, and then promote activation of checkpoints, which results in cell cycle arrest^40^. Whereas the ATR-Chk1 pathway is usually activated by UV light and DNA replication stress, the ATM-Chk2 pathway is mainly activated by the presence of DSBs. Additionally, IR-induced DSBs can also activate the ATR-Chk1 pathway^41,42^ by promoting the formation of a typical dsDNA–ssDNA structure, followed by loading of replication protein A (RPA)^43^. In the current study, we observed that in NSCLC cells IR activated both the ATR-Chk1 and ATM-Chk2 signaling pathways. Nevertheless, among the four kinases, Chk1 was the only one whose phosphorylation level induced by IR was regulated by ERK5, suggesting a critical role of ERK5 in generation of the ssDNA-RPA signaling platform for subsequent recruitment of ATR.

Alteration of cell cycle progression by inducing G2/M phase arrest might be one of the mechanisms underlying radiosensitization^44^. In the current study, we observed that IR-triggered G2/M arrest was enhanced and prolonged following ERK5 knockdown. This may be of great significance to fractionated radiotherapy, because arresting a majority of cells in the most radiosensitive G2/M phase would sensitize them to the next cycle of radiation and increase radiation-induced cell death^45^.

Chk1 has been extensively reported to confer therapeutic resistance in many cancers types, making it a novel target for cancer therapy, especially for potentiating the effectiveness of radiotherapy that induces DNA damage. Since we have found that ERK5 was able to accelerate the phosphorylation and activation of IR-induced Chk1, therapeutic targeting of Chk1 in NSCLC cells with high ERK5 expression might be an effective strategy for overcoming radioresistance. In the in vivo tumor model experiment, ERK5 siRNA combined with low- or high-dose IR therapy strongly inhibited tumor growth and increased the number of TUNEL-positive cells in tumors. Furthermore, decreased staining for CD31 and reduced VEGF expression were also observed, suggesting that a combination of IR and ERK5 siRNA could inhibit tumor progression, primarily by suppressing tumor-related angiogenesis. We observed a significant reduction in body weight in the IR treatment alone group; however, the combination treatment led to a substantial improvement in body weight.

In conclusion, we report for the first time that ERK5 regulates lung cancer cell proliferation, cell cycle progression, DNA damage repair, and angiogenesis, thus promoting lung cancer growth, and protects NSCLC cells exposed to irradiation stress. More importantly, downregulation or pharmacological inhibition of ERK5 sensitized NSCLC cells to IR therapy. These data provide mechanistic insight into the molecular basis of ERK5 signaling in lung cancer radiotherapy, allowing the development of small molecular inhibitors.

## Supplementary information

Supplementary Information
