# Author Correction: Extracellular signal-regulated kinase 5 increases radioresistance of lung cancer cells by enhancing the DNA damage response

**DOI:** 10.1038/s12276-023-01026-9

**Published:** 2023-06-01

**Authors:** Weiwei Jiang, Guanghui Jin, Fangfang Cai, Xiao Chen, Nini Cao, Xiangyu Zhang, Jia Liu, Fei Chen, Feng Wang, Wei Dong, Hongqin Zhuang, Zi-Chun Hua

**Affiliations:** 1 The State Key Laboratory of Pharmaceutical Biotechnology, College of Life Sciences, Nanjing University, Nanjing, PR China; 2 Department of Basic Medical Sciences, Medical College, Xiamen University, Xiamen, PR China; 3 Department of Nuclear Medicine, The Affiliated Nanjing First Hospital, Nanjing Medical University, Nanjing, PR China; 4 Changzhou High-Tech Research Institute of Nanjing University and Jiangsu Target Pharma Laboratories Inc., Changzhou, 213164 PR China

Correction to: *Experimental and Molecular Medicine* 10.1038/s12276-019-0209-3, published online 21 February 2019

After online publication of this article, the authors noticed two errors in the RESULT section.

In the original manuscript, there was a mistake in Fig. 1, in which Fig. 1a and Fig. 1d were misused from another repeated experiment with similar results and the same conclusion. The repeated experiments were actually performed to verify the successful construction of ERK5-overexpression cell line, which was used as experimental material. We sincerely apologize for this and now we would like to provide a new and more representative Fig. 1 as following, in which the Fig. 1a and Fig. 1d have been replaced.
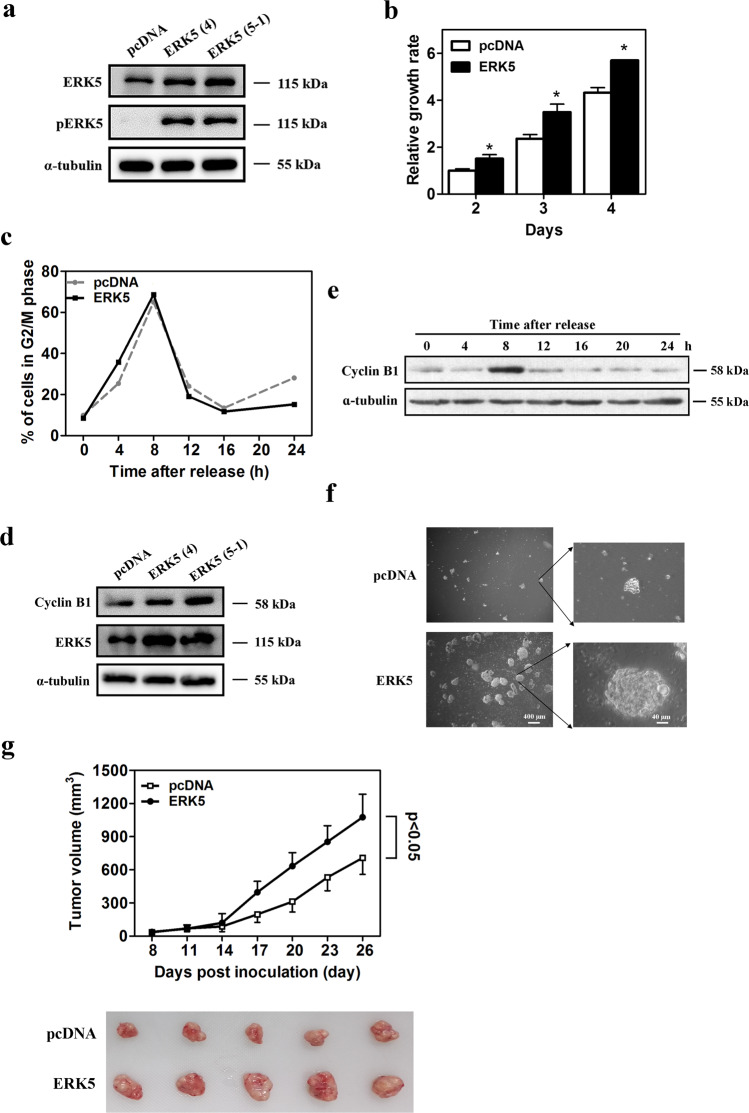


In addition, there might be an inadvertent mistake in Fig. 4 during film scanning for western blotting experiment. We sincerely apologize for this and now we would like to provide a new and more representative Fig. 4 obtained from the repeated experiments with the same results and conclusion.
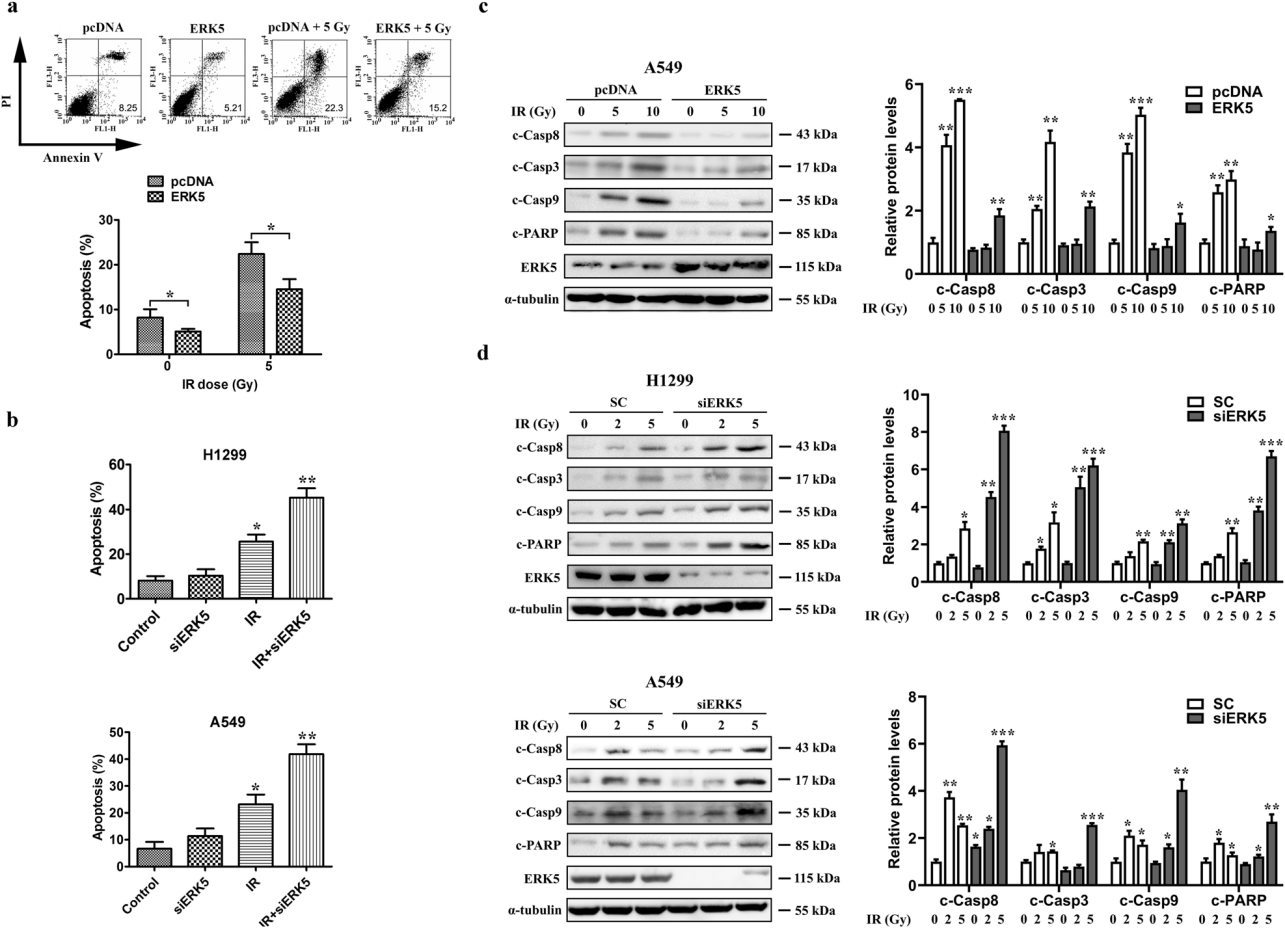


These corrected results do not alter the conclusions of this article. The authors apologize for any inconvenience caused.

The original article has been corrected.

