# Supplementary material for: Extracellular signal-regulated kinase 5 increases radioresistance of lung cancer cells by enhancing the DNA damage response
Source: Exp Mol Med. 2019 Feb 21;51(2):1–20. doi: 10.1038/s12276-019-0209-3 (PMC6389946; doi:10.1038/s12276-019-0209-3)
Supplement: Supplementary file 1 — Supplementary Information [file 12276_2019_209_MOESM1_ESM.pdf]

Extracellular signal-regulated kinase 5 increases radioresistance of lung cancer cells by enhancing the DNA damage response

Weiwei Jiang[1,#], Guanghui Jin[1,2,#], Fangfang Cai[1], Xiao Chen[1], Nini Cao[1], Xiangyu Zhang[1], Jia Liu[1], Fei Chen[3], Feng Wang[3], Wei Dong[1], Hongqin Zhuang[1,*], Zi-Chun Hua[1,4,*]

Supplementary Table S1. Target sequences of shRNA against ERK5.

shRNA	Target sequences
ERK5-1	CCTCATGGAGAGCGACCTACA
ERK5-2	TCTACAGCCTGCAGGCTCTAT
ERK5-3	GCACGAGTATACGCAGGCAAT

Supplementary Table S2. The primers of selected genes for real time PCR.

Gene	Forward primer (5'-3')	Reverse primer (5'-3')
ERK5	GGGAGAAGCGACGACAAGA	CTCACGCTGCAGAGACTCAAT
HIF-1α	ACCTTCATCGGAAACTCCAA	GATTCAAAGTGGCAGACAGCTTA
GRP-78	ACTTGGGGACCACCTATTCCT	AGCATCTTTGGTTGCTTGTCG
VEGF	ACTGGACCCTGGCTTTACT	GCAGGAACATTTACACGTCTG
VEGF-C	TCTGCCAGCAACATTACCACA	CGATTCGCACACGGTCTTC
HSP70	GCGCCCGCGTGATGGATGTG	GGTGCCCGCCTACTTCAACGACTC
β-actin	GAAATCGTGCGTGACATCAAAG	TGTAGTTTCATGGATGCCACAG

Supplementary Figure 1

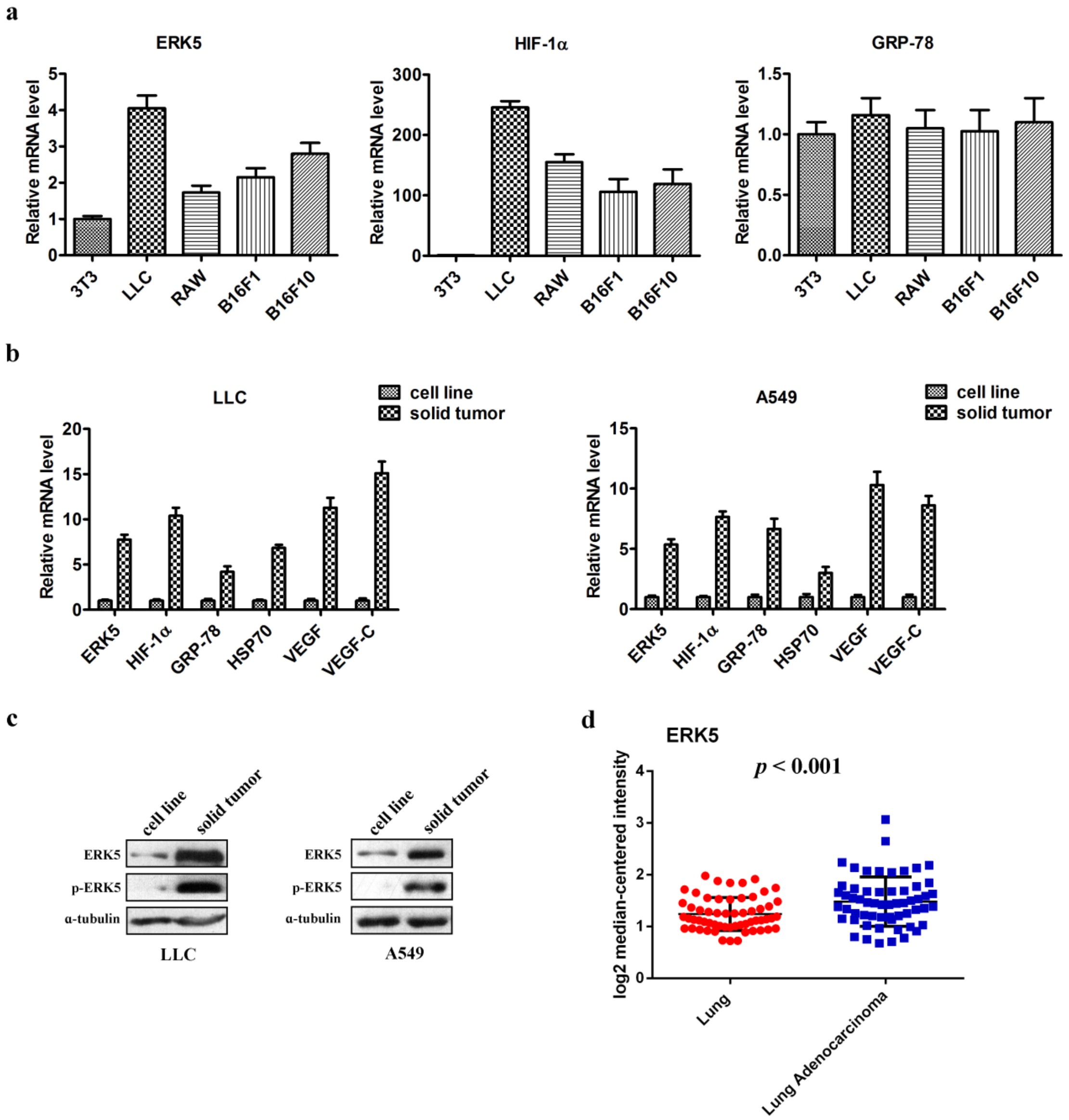

Supplementary Fig. S1 ERK5 expression was up-regulated in lung cancer development. a The mRNA was extracted from several cell lines which were cultured in normal medium conditions. Then ERK5, HIF-1α and GRP-78 mRNA expression levels were detected by qPCR with 28 cycles, using β-actin as internal control. **b** The mRNA was extracted from LLC and A549 solid tumor cells refer to *methods*, and as control, the mRNA was extracted from LLC and A549 cell line which was cultured in normal conditions. Up-regulated ERK5, HIF-1α, HSP70, GRP-78, VEGF and VEGF-C mRNA expression at solid tumor conditions were detected by qPCR with 24

cycles. **c** Whole cell lysate was extracted from LLC (left) and A549 (right) solid tumor cells refer to *methods*, and as control, the protein was extracted from LLC and A549 cell lines which were cultured in normal conditions. Up-regulated ERK5, p-ERK5 protein expression at solid tumor conditions were detected by Western blot analysis. **d** The expression levels of ERK5 in 58 lung adenocarcinoma tumor tissues and 58 normal tissues were obtained from TCGA.

Supplementary Figure 2

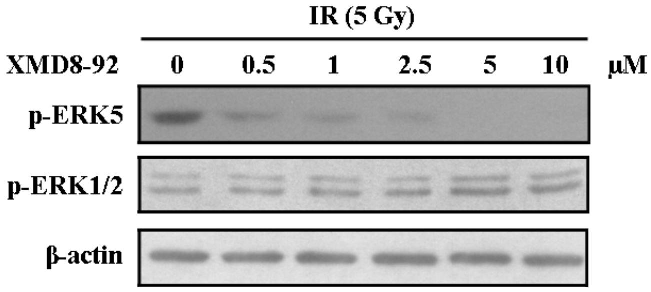

Supplementary Fig. S2 Effects of XMD8-92 on IR-induced phosphorylation of ERK5. A549 cells were treated with 5 Gy X-ray with XMD8-92 at indicated concentrations. 12 h later, whole cell lysates were analyzed by immunoblot with a specific antibody against phospho-ERK5, phospho-ERK1/2, or β-actin, respectively.

Supplementary Figure 3

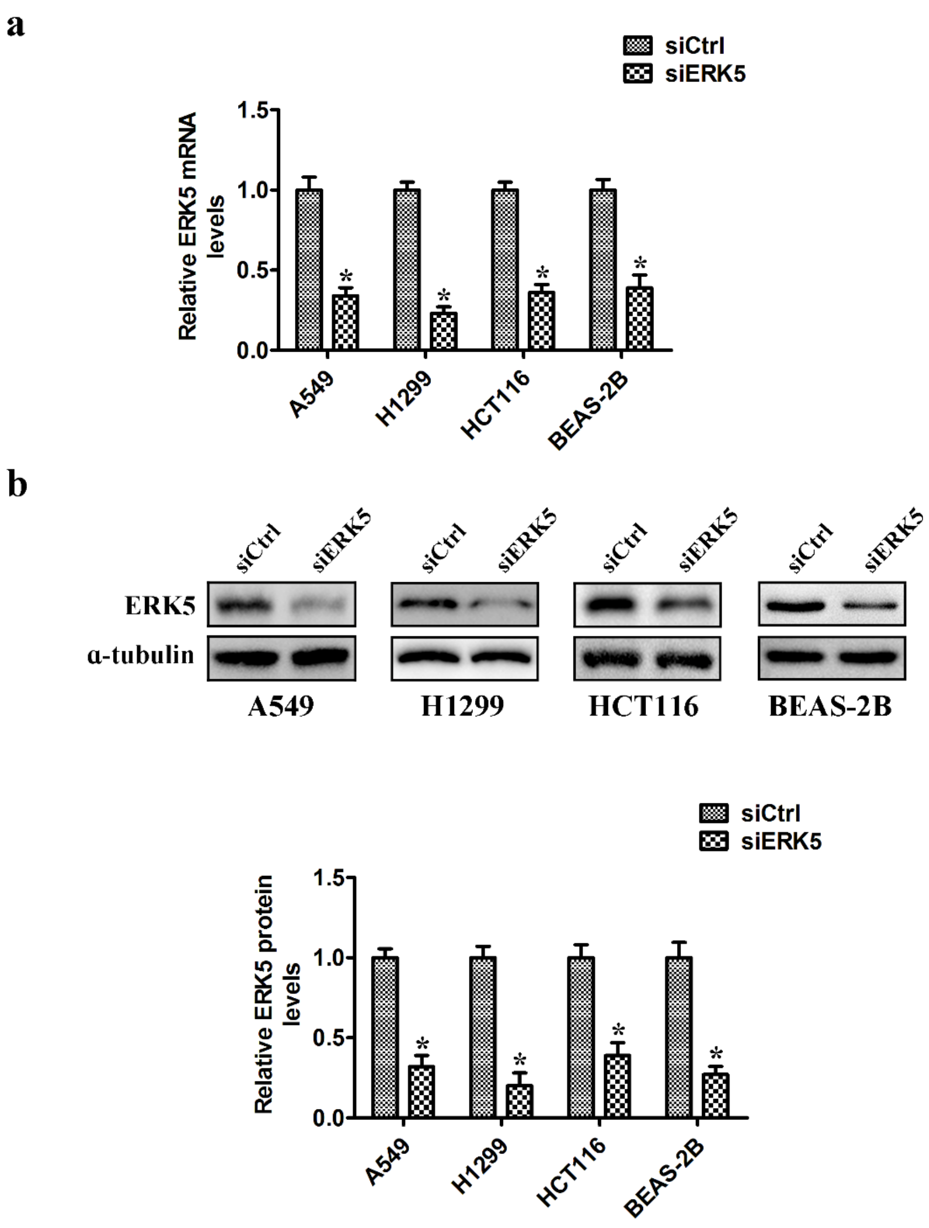

Supplementary Fig. S3 Effect of ERK5 small interfering (si)RNA on ERK5 mRNA and protein. Total RNAs were isolated from A549, H1299, HCT116, and BEAS-2B cells transiently transfected with siCtrl or siERK5 for 48 hours. **a** The relative levels of ERK5 were analyzed by qPCR analysis. **b** Western blotting was used to detect ERK5 protein expression. The levels of ERK5 protein were normalized to

α-tubulin levels in siCtrl-transfected cells. Data are represented as mean ± SD, *$p <$ 0.05 compared with respective control.

Supplementary Figure 4

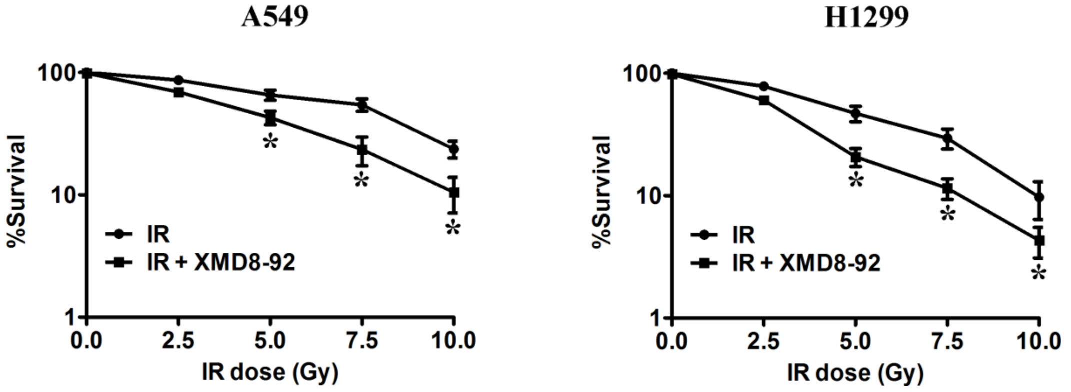

Supplementary Fig. S4 ERK5 inhibitor XMD8-92 radiosensitizes lung cancer cells. Cells were plated at 500 cells per well in a 6-well plate and 24 h later, treated with the indicated dose of IR with or without 5 μM XMD8-92. Cells were then maintained for another 12 days. The colonies were fixed and stained. Number of colonies containing > 50 cells were counted and the percentage of colony formation was determined for each cell line with respect to the non-treated controls. Survival curves for advanced human lung adenocarcinoma cell A549 and H1299 cells are shown. **p* < 0.01 compared with respective control.

Supplementary Figure 5

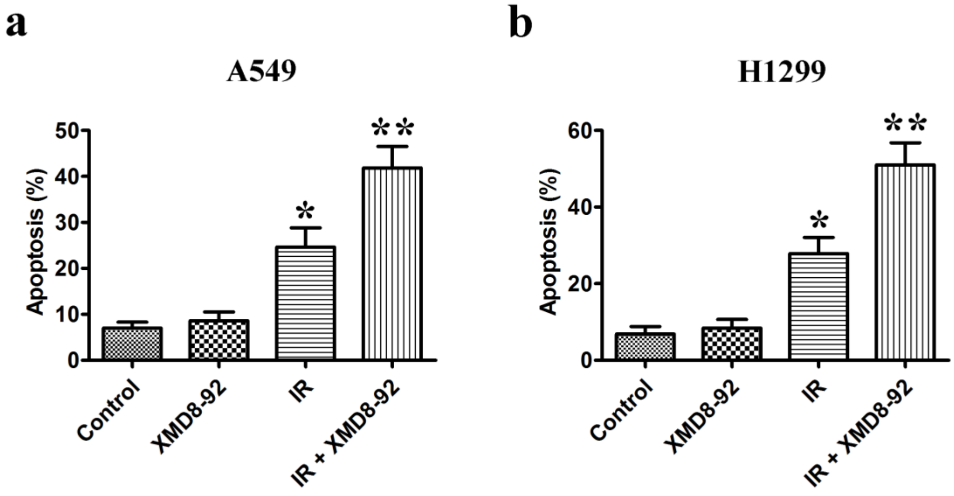

Supplementary Fig. S5 ERK5 inhibitor XMD8-92 triggers IR-induced apoptosis in lung cancer cells. A549 (**a**) and H1299 (**b**) cells were treated by 5 Gy X-ray with or without 5 μM XMD8-92. 16 h later, all cells were harvested for flow cytometry analysis. Annexin V/PI-stained cells were analyzed and the percentage of apoptotic cells was determined. The experiments were carried out independently in triplicate. Data are represented as mean ± SD. *$p < 0.05$ and **$p < 0.01$ compared with respective control.

Supplementary Figure 6

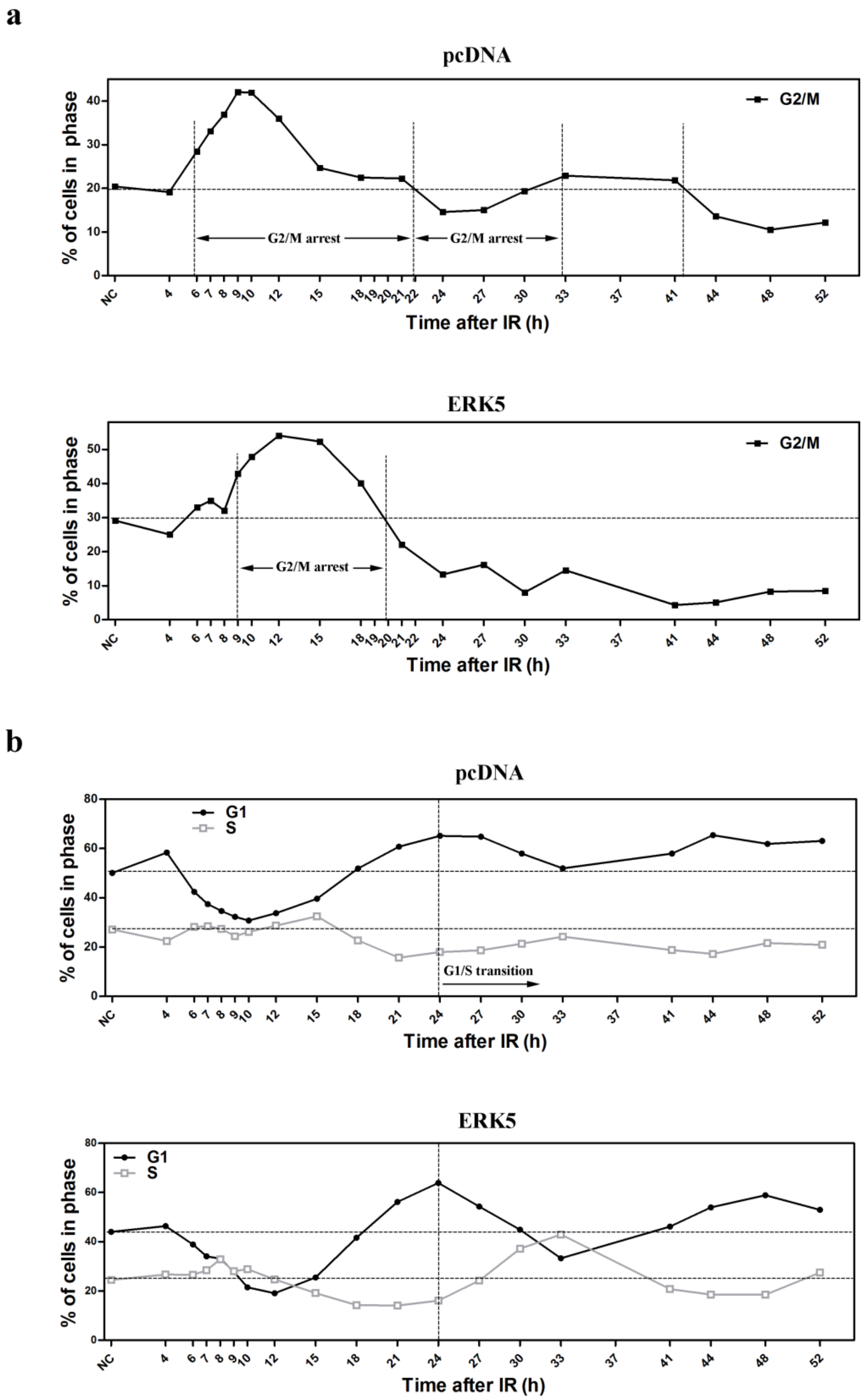

Supplementary Fig. S6 ERK5 promotes cell cycle progression. The stably transfected A549 cells with either empty vector or vector expressing the ERK5 were synchronized by double thymidine block, and harvested at various time points after

release. The cells were stained with PI, following flow cytometry analysis. Cell population in G2/M phase was shown in (**a**), and cell population in G1 and S phase were shown in (**b**).

Supplementary Figure 7

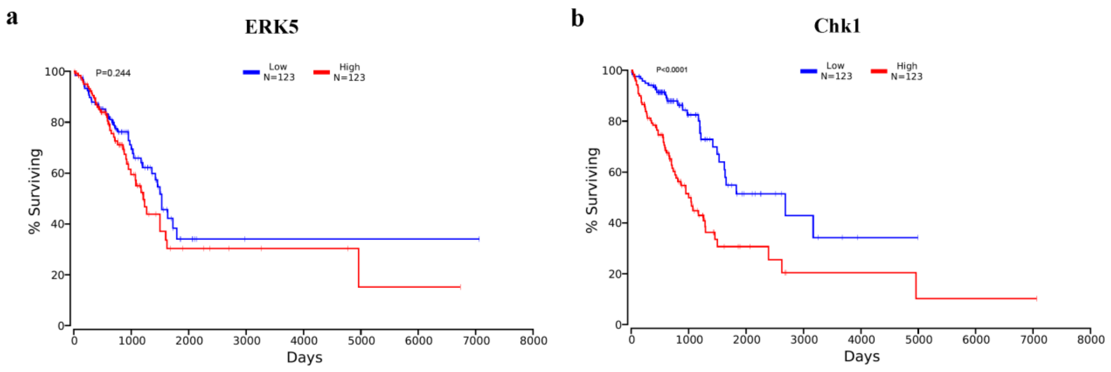

Supplementary Fig. S7 The levels of ERK5 and Chk1 genes correlate with prognosis in lung cancer patients. Kaplan–Meier curves for recurrence free survival were created using the Kaplan-Meier Plotter (www.kmplot.com) with lung cancer patients classified according to high and low ERK5 (**a**) and Chk1 (**b**) gene expression. Hazard ratio (with 95% confidence interval) and log-rank *p* values were calculated.

Supplementary Figure 8

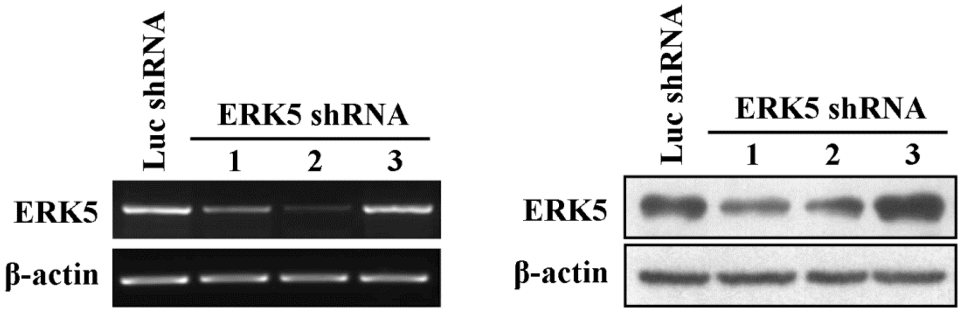

Supplementary Fig. S8 Effect of ERK5 shRNA on ERK5 mRNA and protein. Reverse transcription-polymerase chain reaction (**a**) and western blotting (**b**) were used to detect ERK5 mRNA abundance and protein expression. Mouse lewis lung cancer cell line LLC was used.
